# Sources of Variation in Cardiovascular Care Cascades

**DOI:** 10.1001/jamahealthforum.2026.0491

**Published:** 2026-04-03

**Authors:** Annabel Z. Wang, Divya Shanmugam, Sanjay Divakaran, Emma Pierson, Michael L. Barnett

**Affiliations:** 1 Department of Medicine, Brigham and Women’s Hospital, Boston, Massachusetts; 2 Department of Medicine, Harvard Medical School, Boston, Massachusetts; 3 Department of Computer Science, Cornell Tech, New York, New York; 4 Department of Electrical Engineering and Computer Science, Massachusetts Institute of Technology, Cambridge; 5 Division of Cardiovascular Medicine, Brigham and Women’s Hospital, Boston, Massachusetts; 6 Department of Electrical Engineering and Computer Sciences, University of California, Berkeley; 7 Department of Health Services Policy and Practice, Brown University School of Public Health, Providence, Rhode Island

## Abstract

**Importance:**

Variation in cardiovascular care completion is well documented. However, less is known about differences originating from earlier, intermediate stages such as ordering or scheduling of testing or referrals, despite their role as key prerequisites for care access.

**Objective:**

To examine the care cascades for coronary artery disease (CAD) after emergency department (ED) visits and to identify the specific stages at which variation emerges for CAD testing and cardiology referrals.

**Design, Setting, and Participants:**

This was a retrospective cohort study using data and metadata from electronic health records from a large multicenter health system. Participants were adult patients with established primary care and no history of ischemic heart disease or cardiology care who presented to an ED from January 1, 2020, to June 30, 2022, and underwent a troponin test, a proxy for clinically suspected myocardial ischemia. Variation in cardiovascular follow-up care (CAD testing and cardiology referrals) was identified and analyzed. Analyses were restricted to patients with above-median electrocardiogram (ECG)-derived ischemia risk scores to enrich for higher likelihood of benefit from cardiovascular follow-up.

**Main Outcomes and Measures:**

Receipt of an order for CAD testing (stress tests, coronary computed tomography angiography) or cardiology referral, scheduling of the service, and completion within 6 months. Outcomes were compared by insurance type, race and ethnicity, language, and sex using multivariable logistic regression adjusted for demographic characteristics, clinical factors, and ECG-derived cardiovascular risk.

**Results:**

Among 16 475 patients with an ED visit (median [IQR] age, 67.4 [54.9-77.9] years; 36% female and 64% male individuals) and elevated cardiovascular risk, marked variation in follow-up care emerged. Compared to commercially insured patients, those with Medicare dual or disabled coverage had lower adjusted odds of completing CAD testing (adjusted odds ratio [aOR], 0.45; 95% CI, 0.36-0.56) and cardiology referrals (aOR, 0.47; 95% CI, 0.39-0.57); similar patterns were seen for Medicaid coverage. Patients whose primary language was not English were less likely to complete either service (CAD testing aOR, 0.77; 95% CI, 0.61-0.98; referral aOR, 0.75, 95% CI, 0.61-0.92), and female patients had lower adjusted odds of completing CAD testing (aOR, 0.86; 95% CI, 0.77- 0.96). Adjusted differences by race and ethnicity were modest. Variation was primarily associated with ordering differences and with additional scheduling barriers for select groups. Once scheduled, completion rates exceeded 75%, without differences between groups.

**Conclusions and Relevance:**

This retrospective cohort study found that among patients who visited the ED with elevated ischemic risk, attrition in follow-up care was concentrated early in care cascades and most pronounced among those with noncommercial health insurance. This stepwise analytic framework offers a novel, reproducible approach for health systems to identify where and for whom care gaps arise, which can enable targeted interventions to improve equity and efficiency.

## Key Points

**Question:**

What stages within cardiovascular care cascades account for variation in follow-up care after emergency department (ED) visits for patients at elevated cardiovascular risk?

**Findings:**

In this retrospective cohort study of 16 475 patients with an ED visit and elevated cardiovascular risk, variation in follow-up care was concentrated in the early cascade stages, particularly ordering and scheduling of testing and/or referrals; once scheduled, completion rates were high. Differences were most pronounced by health insurance type, patient language, and sex.

**Meaning:**

Mapping variation across discrete care cascade stages can identify actionable intervention points to improve equity and efficiency in health care.

## Teaser

This cohort study identifies variations in the care cascade for patients in need of coronary artery disease testing and cardiology referral after an emergency department visit.

## Introduction

Early detection and management of cardiovascular disease are essential for improving clinical outcomes.^1,2^ Despite advances in prevention and treatment, variation in the receipt of cardiovascular care persists, contributing to worse outcomes for historically disadvantaged populations.^3,4,5,6^ These differences are well-documented in access to treatment end points, such as evidence-based medications or coronary revascularization.^3,6,7,8,9^ However, less attention has been given to inequities in earlier, intermediate stages of care. These stages are often overlooked but are prerequisites for patients to ultimately receive an end point service.

For patients presenting to emergency departments (EDs) with suspected myocardial ischemia, further evaluation with diagnostic testing or cardiology referral is often recommended based on risk stratification. For example, cardiac stress testing or coronary computed tomography angiography testing (CCTA)—referred to collectively as coronary artery disease (CAD) testing—is recommended for patients at intermediate or higher cardiovascular risk without known CAD by recent chest pain guidelines.^10^ However, successful completion of these diagnostic pathways depends on a sequence of intermediate stages, such as order placement and scheduling, followed by completion.

These intermediate stages require complex coordination and multiple points of contact and engagement from both patients and clinicians. Thus, they represent critical junctures where differences in follow-up may arise. Barriers such as limited transportation, variable internet access, and inflexible work schedules increase the risk of loss of follow-up and disproportionately impact low-income and minority patients.^11,12,13,14,15^ However, the relative contribution of differences at each intermediate step in cardiovascular care cascades remains poorly understood. Closer examination of these stages could provide a more precise mechanistic understanding of when and why variation in cardiovascular outcomes emerge.

Leveraging detailed electronic health record (EHR) metadata capturing order entry, scheduling, and appointment completion time stamps, we examined differences in the completion of intermediate stages in cardiovascular follow-up care cascades. In this study, we focused on CAD testing and cardiology referrals after ED visits. We restricted the cohort to patients with an ordered troponin test, a proxy for clinical suspicion of myocardial ischemia; we further enriched for patients at higher cardiovascular risk using an ECG-derived risk score. By dissecting differential attrition step by step, we aimed to uncover actionable insights into how cardiovascular care differences manifest and provide a foundation for developing targeted interventions to improve health equity on a larger scale.

## Methods

The institutional review board of Mass General Brigham approved the study and waived informed consent under the revised Common Rule (45 CFR § 46.104[d]). This study followed the Strengthening the Reporting of Observational Studies in Epidemiology (STROBE) reporting guideline. Additional methodological details are available in eMethods in Supplement 1.

### Data Collection and Study Population

This study used comprehensive EHR data, including time-stamped clinical event metadata, from a large academic health care system comprising 11 hospitals and hundreds of affiliated practices. We identified all adult patients presenting to the health system’s emergency departments (EDs) between 2016 and 2022 with a troponin test ordered. A troponin test order served as a proxy for clinical concern for myocardial ischemia, capturing both typical and atypical presentations while reducing bias from incomplete or inconsistent symptom documentation. Patients were required to have an established primary care physician within the health system to minimize missing data attributable to care received outside the health system.

To focus on patients with minimal prior cardiovascular evaluation, we excluded patients with documented diagnoses of ischemic heart disease or established cardiology care before the index ED visit. Patients were included regardless of ED disposition, such as hospital admission, ED observation, or home.

### Electrocardiogram-Derived Cardiovascular Risk Score

We developed a model using raw 12-lead electrocardiogram (ECG) waveforms to estimate individual risk of abnormal cardiac perfusion. Using a dilated convolutional neural network architecture fine-tuned from PreOpNet,^16^ the model was trained on patients who underwent nuclear stress testing between 2016 and 2019, with abnormal perfusion confirmed by imaging. Machine-learning ECG models detect subtle ischemic risk signatures often missed by visual interpretation. Multicenter validation studies have demonstrated that these models outperform conventional clinical risk scores (eg, HEART score) and troponin-only strategies for diagnostic discrimination and risk classification.^17,18^

This model substantially outperformed models relying solely on demographic characteristics or preprocessed ECG features. The resulting ECG-derived risk score was intended to provide a standardized, objective cardiovascular risk measure while reducing bias from differential availability of traditional clinical risk data, such as lipid markers. Each patient presenting between 2020 and 2022 was assigned a cardiovascular risk score based on the ECG performed on or most recently prior to the index ED visit.

To enrich for patients more likely to benefit from cardiovascular follow-up, we restricted the cohort to patients with an ECG-derived risk score above the sample median (eFigure 1 in Supplement 1). In a held-out validation set from the 2016 to 2019 sample, patients above this threshold were 2.8-fold more likely to have abnormal cardiac perfusion on stress test imaging compared with those below the median, reflecting higher pretest probability of clinically significant ischemia; across all patients with an ED troponin test ordered, there was a 29.6% baseline abnormal perfusion rate within 6 months. This restriction does not imply that all included patients required further cardiovascular testing or referral but establishes a more clinically comparable risk stratum to evaluate whether differences persist after adjusting for measured risk factors. By combining troponin ordering with above-median ECG-derived ischemia risk restrictions and adjusting for relevant covariates, we aimed to compare patient subgroups where similar clinical consideration for further cardiovascular evaluation would be plausible.

### Outcome Measures: Intermediate and End Point Cascade Stages

The primary study outcomes were completion of key intermediate stages in cardiovascular care cascades within 6 months of an index ED visit (eFigure 2 in Supplement 1). The CAD testing end point included cardiac stress testing or CCTA. The cardiology referral end point included office visits within a cardiology department. The cascade stages were defined as (1) receipt of a relevant order (for CAD testing or cardiology referral); (2) scheduling of the order; and (3) completion of the scheduled service. Intermediate stage completion was determined using EHR time stamps, identifying the earliest completion date for each stage (ordering, scheduling, completion) per patient following the ED visit, if present. Patients receiving invasive coronary angiography during the index episode were excluded from CAD testing analyses to focus on lower- to medium-acuity presentations where discretionary use of noninvasive testing may contribute to variation.

### Study Variables

Patient demographic characteristics, including age, sex, self-reported race and ethnicity, and primary language were obtained from the EHR. Primary health insurance payer was determined using the patient’s effective insurance at ED arrival. Comorbidity burden was calculated as the total distinct *International Statistical Classification of Diseases and Related Health Problems, Tenth Revision (ICD-10) *codes tied to professional billing charges in the preceding year. Troponin levels were extracted as the highest value linked to and resulting within 72 hours of the index ED visit, then categorized as within normal limits; 1 to 3 times the upper limit of normal; and more than 3 times the upper limit of normal. Kidney function via estimated glomerular filtration rate (eGFR) value was obtained from the year prior to and including the ED visit.

### Statistical Analyses

Logistic regression models estimated differences in the completion of each cascade step across patient characteristics, including race and ethnicity, health insurance type, primary language, and sex. For each characteristic of interest, we fit separate models estimating differences in completing a given step conditional on having completed the prior step in the care cascade, and unconditional completion of the full cascade. Models were adjusted for age, sex, ECG-derived cardiovascular risk score, comorbidity burden, magnitude of troponin elevation, and inpatient admission status.

### Sensitivity Analyses

To address potential residual confounding by clinical severity, we conducted sensitivity analyses. First, we applied alternative definitions of high-risk status, including patients with a troponin level exceeding the assay-specific upper limit of normal and those meeting American College of Cardiology/American Heart Association chest pain guideline criteria for further testing (HEART score criterion incorporating age 65 years or older and markedly elevated troponin, qualifying as intermediate risk or higher).^10,19,20^ Second, we excluded patients with reduced kidney function (eGFR <60 mL/min/1.73 m^2^), as these patients may have elevated troponin without coronary disease. To account for site-specific, temporal, and additional sociodemographic variation, we applied fixed-effects models using ED department, month-year, and patient postal code indicators. We calculated E-values to evaluate robustness of key associations to potential unmeasured confounding (eTable 3 in Supplement 1).^21^ Finally, inverse probability of censoring weighting (IPCW) sensitivity analyses assessed potential bias from censoring due to death before outcome measurement (eTable 4 in Supplement 1).

Statistical tests were 2-tailed and *P* = .05 was considered to be statistically significant. Data were analyzed from June 7, 2023, to December 22, 2025, using R, version 4.4.2 (R Foundation for Statistical Computing).

## Results

The analysis included a total of 16 475 adult patients (median [IQR] age, 67.4 [54.9-77.9] years; 36.0% female and 64.0% male individuals) who presented to the EDs from January 1, 2020, to June 30, 2022, and met the cohort criteria, which included enrichment for elevated cardiovascular risk, no prior cardiovascular evaluation, and an ordered troponin test. Regarding race and ethnicity, 4.2% were Hispanic or Latino, 5.8% were Black (not Hispanic), and 81.2% were White (not Hispanic). Regarding health insurance, 32.2% of the participants had commercial health insurance coverage, 10.7% had Medicaid coverage, and 10.8% had Medicare dual or disabled coverage. Additional characteristics are detailed in Table 1.

**Table 1.** Characteristics of Patients With an Emergency Department (ED) Visit, a Troponin Test Ordered, and Above-Median Electrocardiogram-Derived Cardiovascular Risk Score

Characteristic	Patients, No. (%)^a^
Total, No.	16 475
Age, median (IQR), y	67.4 (54.9-77.9)
Sex	
Female	5926 (36.0)
Male	10 549 (64.0)
Health insurance payer	
Commercial	5299 (32.2)
Medicare	7480 (45.4)
Medicaid	1759 (10.7)
Medicare−dual or disabled	1773 (10.8)
Race and ethnicity^b^	
Black	953 (5.8)
Hispanic or Latino	695 (4.2)
White	13 378 (81.2)
Asian and other^c^	1449 (8.8)
Primary language	
English	15 169 (92.1)
Spanish	440 (2.7)
Other	866 (5.3)
Inpatient admission at ED visit	9409 (57.1)
Death within 6 mo of ED discharge	1135 (6.9)

^a^ Percentages are rounded and may not add up to 100%.

^b^ Race and ethnicity were obtained from the electronic health record and reflect patient self-identification at registration. If not included in Hispanic or Latino, patient was not of Hispanic or Latino ethnicity.

^c^ Asian and other includes American Indian or Alaska Native, Asian, Native Hawaiian or Other Pacific Islander, other, or declined.

### Unadjusted Completion of Cardiovascular Follow-Up Services

**Figure 1.**
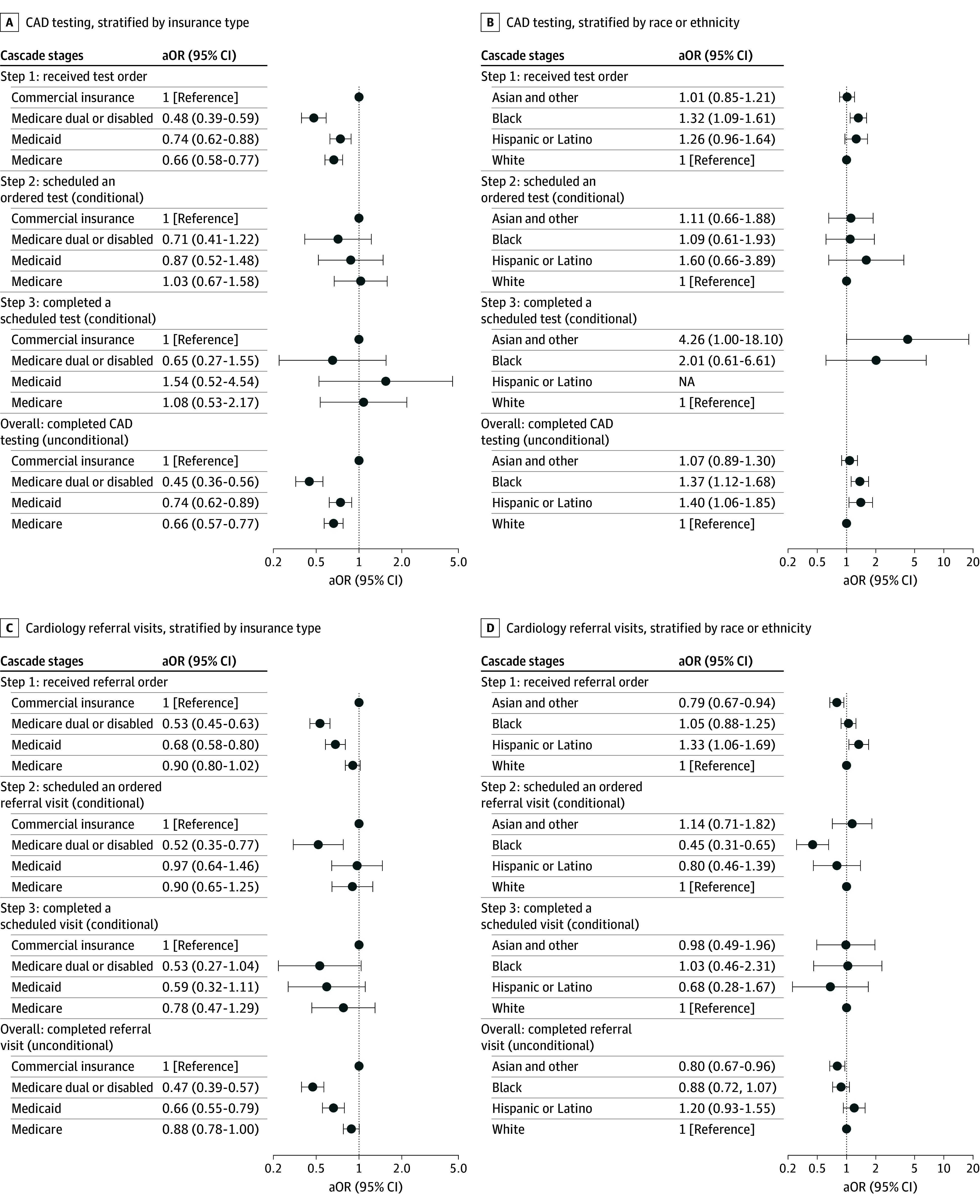
Forest Plot of Adjusted Completion of End Point and Intermediate Stages in Cardiovascular Follow-Up Care Coronary artery disease (CAD) testing collectively refers to cardiac stress testing or coronary computed tomography angiography. Subgroup with the highest unadjusted completion was selected as the reference group to facilitate comparison. For “CAD testing step 3: completed a scheduled test (conditional),” all patients in the Hispanic or Latino subgroup completed the step, which precluded model estimation; therefore, this subgroup is not displayed (panel D). Regarding race and ethnicity, these data were obtained from the electronic health record and reflect patient self-identification at registration; if not included in Hispanic or Latino, patient was not of Hispanic or Latino ethnicity; and Asian and other includes American Indian or Alaska Native, Asian, Native Hawaiian or Other Pacific Islander, other, or declined. Error bars represent 95% CIs and aORs indicate adjusted odds ratios.

Descriptive, unadjusted completion rates of cardiovascular follow-up within 6 months of the index ED visit varied by insurance type, race and ethnicity, primary language, and sex (Table 2). Patients with Medicare dual or disabled insurance, Medicaid insurance, or a non-English primary language had lower completion rates than reference subgroups (commercial insurance; English). Female patients had lower unadjusted CAD testing completion than male patients. These descriptive differences were primarily driven by variation in ordering; conditional on receiving an order, completion rates were high (≥79.9% for CAD testing and ≥71.9% for cardiology referrals across all groups). Unadjusted rates do not account for clinical or demographic covariates; adjusted analyses (Figure 1 and Table 3) provide a more rigorous assessment of differences and serve as the primary basis for inference.

**Table 2.** Unadjusted Completion Rates of Cardiovascular Follow-Up Stages After Emergency Department Visit With Troponin Test Ordered and Above-Median Electrocardiogram-Derived Cardiovascular Risk

Subgroup	No. of patients	Patients in subgroup completing each stage, %							
		CAD testing				Cardiology referral visit			
		Received order	Scheduled	Completed	% Completed (of ordered)	Received order	Scheduled	Completed	% Completed (of ordered)
Total patients	16 475	13.6	12.1	11.6	84.8	21.0	18.6	17.7	83.9
Health insurance payer									
Commercial	5299	17.3	15.6	14.9	86.3	19.6	17.0	16.1	82.4
Medicare	7480	12.4	10.8	10.3	83.7	25.0	22.8	21.8	87.0
Medicaid	1759	12.8	11.4	11.2	87.4	14.8	12.4	11.5	77.7
Medicare−dual or disabled	1773	9.4	8.0	7.5	79.9	15.7	12.7	11.9	75.9
Race and ethnicity^a^									
Asian and other^b^	1449	13.0	11.7	11.5	88.5	14.9	13.2	12.5	83.8
Black	953	16.7	14.9	14.5	86.8	20.6	15.5	14.8	71.9
Hispanic or Latino	695	14.4	13.2	13.2	91.4	18.8	15.5	14.5	77.1
White	13 378	13.4	11.9	11.3	83.9	21.8	19.6	18.6	85.0
Primary language									
English	15 169	13.8	12.2	11.7	84.8	21.4	19.0	18.0	84.0
Other than English	1306	11.8	10.3	10.1	85.5	16.4	14.0	13.6	82.7
Sex									
Female	5926	12.2	10.7	10.2	83.3	21.3	18.8	17.9	83.7
Male	10 549	14.4	12.9	12.3	85.5	20.9	18.5	17.5	84.0

Abbreviation: CAD, coronary artery disease.

CAD testing collectively refers to cardiac stress testing or coronary computed tomography angiography (eMethods in Supplement 1).

^a^ Race and ethnicity were obtained from the electronic health record and reflect patient self-identification at registration. If not included in Hispanic or Latino, patient was not of Hispanic or Latino ethnicity.

^b^ Asian and other includes American Indian or Alaska Native, Asian, Native Hawaiian or Other Pacific Islander, other, or declined.

**Table 3.** Adjusted Odds Ratios for Completion of Cardiovascular Follow-Up Care End Points and Intermediate Stages

Subgroup	No. of patients (N = 16 475)	Adjusted ORs (95% CIs)							
		CAD testing				Cardiology referral visit			
		Received test order	Scheduled an ordered test	Completed a scheduled test	Overall: completed test (unconditional)	Received referral order	Scheduled an ordered referral	Completed a scheduled visit	Overall: completed visit (unconditional)
Insurance payer									
Commercial	5299	1 [Reference]	1 [Reference]	1 [Reference]	1 [Reference]	1 [Reference]	1 [Reference]	1 [Reference]	1 [Reference]
Medicare	7480	0.66 (0.58-0.77)	1.03 (0.67-1.58)	1.08 (0.53-2.17)	0.66 (0.57-0.77)	0.90 (0.80-1.02)	0.90 (0.65-1.25)	0.78 (0.47-1.29)	0.88 (0.78-1.00)
Medicaid	1759	0.74 (0.62-0.88)	0.87 (0.52-1.48)	1.54 (0.52-4.54)	0.74 (0.62-0.89)	0.68 (0.58-0.80)	0.97 (0.64-1.46)	0.59 (0.32-1.11)	0.66 (0.55-0.79)
Medicare−dual or disabled	1773	0.48 (0.39-0.59)	0.71 (0.41-1.22)	0.65 (0.27-1.55)	0.45 (0.36-0.56)	0.53 (0.45-0.63)	0.52 (0.35-0.77)	0.53 (0.27-1.04)	0.47 (0.39-0.57)
Race and ethnicity^a^									
Asian and other^b^	1449	1.01 (0.85-1.21)	1.11 (0.66-1.88)	4.26 (1.00-18.10)	1.07 (0.89-1.30)	0.79 (0.67-0.94)	1.14 (0.71-1.82)	0.98 (0.49-1.96)	0.80 (0.67-0.96)
Black	953	1.32 (1.09-1.61)	1.09 (0.61-1.93)	2.01 (0.61-6.61)	1.37 (1.12-1.68)	1.05 (0.88-1.25)	0.45 (0.31-0.65)	1.03 (0.46-2.31)	0.88 (0.72-1.07)
Hispanic or Latino	695	1.26 (0.96-1.64)	1.60 (0.66-3.89)	NA^c^	1.40 (1.06-1.85)	1.33 (1.06-1.69)	0.80 (0.46-1.39)	0.68 (0.28-1.67)	1.20 (0.93-1.55)
White	13 378	1 [Reference]	1 [Reference]	1 [Reference]	1 [Reference]	1 [Reference]	1 [Reference]	1 [Reference]	1 [Reference]
Primary language									
English	15 169	1 [Reference]	1 [Reference]	1 [Reference]	1 [Reference]	1 [Reference]	1 [Reference]	1 [Reference]	1 [Reference]
Other than English	1306	0.80 (0.64-1.00)	0.83 (0.45-1.54)	0.68 (0.20-2.36)	0.77 (0.61-0.98)	0.72 (0.60-0.87)	0.93 (0.57-1.53)	1.98 (0.78-5.04)	0.75 (0.61-0.92)
Sex									
Female	5926	0.88 (0.80-0.98)	0.83 (0.62-1.12)	1.03 (0.61-1.71)	0.86 (0.77-0.96)	1.01 (0.93-1.10)	0.88 (0.69-1.11)	0.80 (0.56-1.14)	0.99 (0.90-1.08)
Male	10 549	1 [Reference]	1 [Reference]	1 [Reference]	1 [Reference]	1 [Reference]	1 [Reference]	1 [Reference]	1 [Reference]

Abbreviations: CAD, coronary artery disease; NA, not applicable; ORs, odds ratios.

CAD testing collectively refers to cardiac stress testing or coronary computed tomography angiography.

^a^ Race and ethnicity were obtained from the electronic health record and reflect patient self-identification at registration. If not included in Hispanic or Latino, patient was not of Hispanic or Latino ethnicity.

^b^ Asian and other includes American Indian or Alaska Native, Asian, Native Hawaiian or Other Pacific Islander, other, or declined.

^c^ For “CAD testing stage 3: completed a scheduled test (conditional),” all patients in the Hispanic or Latino subgroup completed the stage, which precluded model estimation; therefore, this estimate is not displayed.

### Adjusted Completion of Cardiovascular Follow-Up Services

After adjusting for ECG-derived cardiovascular risk, age, comorbidities, troponin magnitude, admission status, and sociodemographic covariates, substantial differences in cardiovascular follow-up persisted across multiple subgroups (Figure 1 and Table 3; eTable 2 in Supplement 1 for adjusted probabilities). Compared with commercially insured patients, those with Medicare dual or disabled insurance had lower odds of completing CAD testing (aOR, 0.45; 95% CI, 0.36 to 0.56) or cardiology referral visits (aOR, 0.47; 95% CI, 0.39 to 0.57). Similarly, Medicaid patients had lower adjusted odds of completion for both end points (CAD testing aOR, 0.74; 95% CI, 0.62 to 0.89; cardiology referral aOR, 0.66; 95% CI, 0.55 to 0.79). Non-English primary language was independently associated with lower completion of both end points, and female patients had lower adjusted odds of completing CAD testing. Asian and other non-Hispanic patients had lower odds of completing cardiology referrals compared with White patients (aOR, 0.80; 95% CI, 0.67 to 0.96).

Temporal analyses revealed that CAD testing differences emerged rapidly following the ED visit and remained stable for 6 months (eTable 1 in Supplement 1). For cardiology referrals, differences in completion were less evident within the first 2 weeks but widened by 30 days and persisted thereafter.

### Completion Rates for Intermediate Cascade Stages 

While overall differences in service completion were evident, not all intermediate steps contributed equally to these gaps. Differences in receiving orders were the primary drivers of variation by insurance type, primary language, and sex (Figure 1 and Table 3). For example, Medicare dual or disabled patients were less likely to receive CAD testing orders than commercially insured patients (aOR, 0.48; 95% CI, 0.39 to 0.59) as were all other noncommercially insured patient groups. Similarly, Asian and other non-Hispanic patients were less likely to receive cardiology referral orders (aOR, 0.79; 95% CI, 0.67 to 0.94).

For a subset of patients, scheduling barriers also contributed to variation. Conditional on receiving a referral order, Medicare dual or disabled patients were less likely to schedule cardiology visits (aOR, 0.52; 95% CI, 0.35 to 0.77). Black patients were also less likely to schedule ordered cardiology visits (aOR, 0.45; 95% CI, 0.31 to 0.65), although this did not translate into differences in overall referral completion. Conditional on services being scheduled, completion rates were comparable across all subgroups.

### Sensitivity Analyses

**Figure 2.**
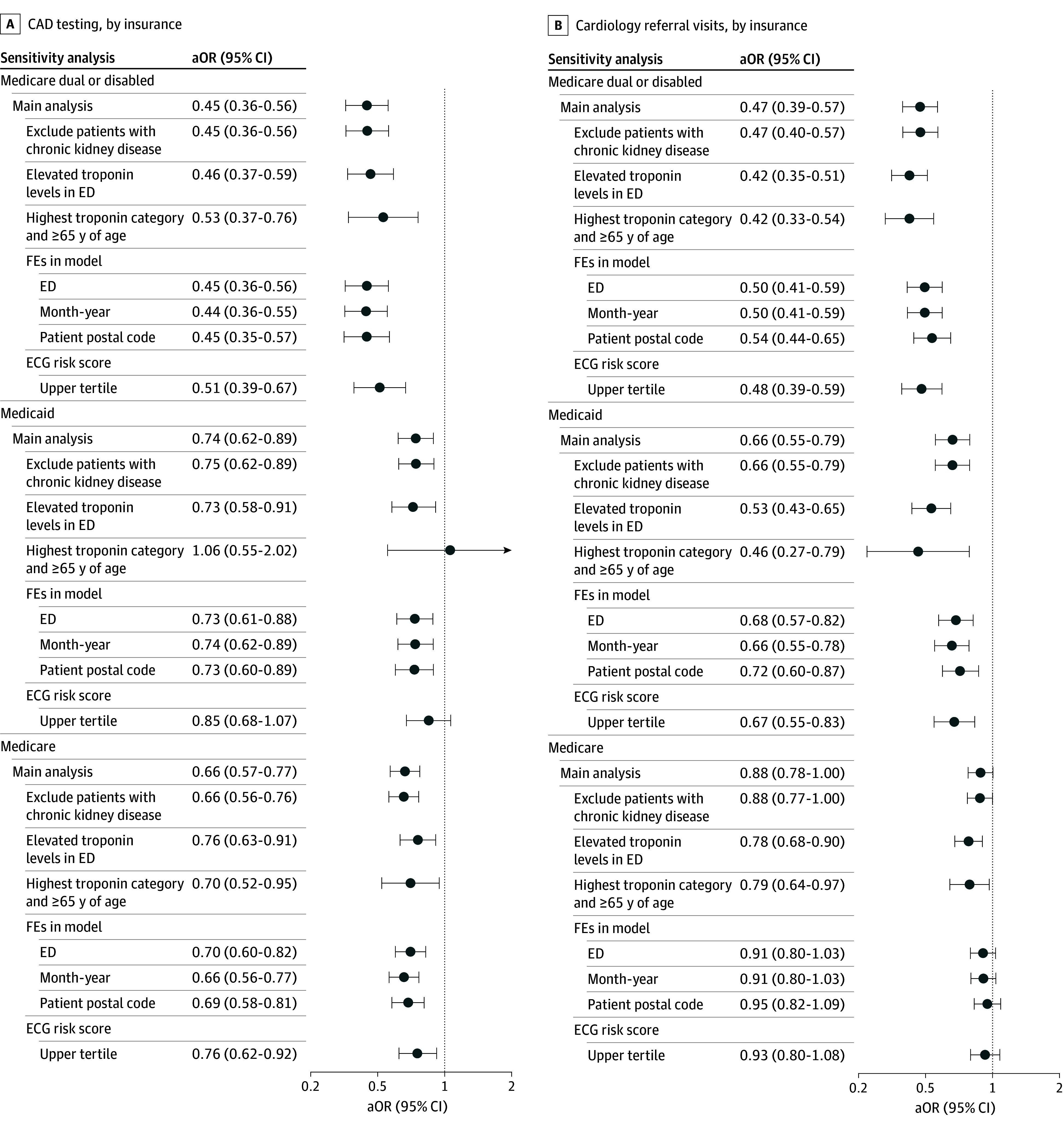
Forest Plot of Sensitivity Analyses for Follow-Up Care Cascade Completion After Emergency Department (ED) Visits, Stratified by Insurance Type Models were adjusted for electrocardiogram-derived cardiovascular risk, age, sex, comorbidities, troponin magnitude, and admission status. For patients aged 65 years or older with troponin levels in the highest category included 110 Medicaid-insured patients due to near-universal additional Medicare coverage in this age group; this small sample likely contributed to the wider 95% CIs for this subgroup. Regarding race and ethnicity, these data were obtained from the electronic health record and reflect patient self-identification at registration; if not included in Hispanic or Latino, patient was not of Hispanic or Latino ethnicity; and Asian and other includes American Indian or Alaska Native, Asian, Native Hawaiian or Other Pacific Islander, other, or declined. aOR indicates adjusted odds ratio; ECG, electrocardiogram; FE, fixed effects.

Model estimates remained stable after excluding patients with reduced kidney function and adjusting for site and temporal factors (Figure 2 and eFigure 3 in Supplement 1). Alternative definitions of high-risk clinical status yielded largely consistent results; reduced referral visit completion among Medicare patients emerged with restriction to higher risk strata. IPCW adjustment for death censoring produced nearly identical results, supporting robustness to potential follow-up bias. Overall, sensitivity analyses reflected persistent subgroup variation (Figure 2; eMethods, eFigure 3, and eTable 4 in Supplement 1).

## Discussion

In this large cohort study using detailed EHR metadata, we identified substantial variation in cardiovascular follow-up care after ED visits. Differences by insurance type, primary language, sex, and race and ethnicity persisted after adjustment for clinical characteristics and ECG-derived cardiovascular risk. Using a stepwise analytic framework, we found that early-stage disruptions in care cascades were key drivers of variation in end point completion, particularly at ordering, and sometimes scheduling, stages. Variation was most pronounced and consistent by insurance type, followed by language and sex. Adjusted racial and ethnic differences were smaller in magnitude, although still present for select services. Notably, differences were not uniform; they varied by cardiovascular service and patient subgroup, underscoring that there is no one-size-fits-all intervention point or approach to improve equitable access to care.

Although variation arises from a complex interplay of clinician, patient, and system factors, clarifying the intermediate stages that most contribute can provide insight into underlying mechanisms. Our findings that lower ordering rates drove differences for noncommercially insured patients, non-English speakers, and female patients may reflect clinician biases, operational constraints, or both. For example, clinicians may place fewer orders for patients anticipated to face financial or logistical barriers, perpetuating a cycle of reduced engagement and incomplete care. Administrative burdens, such as prior authorization, copay structures, or network limitations may further complicate ordering decisions, particularly for noncommercial beneficiaries.^22,23^ These system-level factors represent actionable targets to reduce inequities. Ordering differences may also stem from biased clinical risk perceptions or patient-clinician communication challenges. Prior research has documented higher risk of treatment bias with underutilization of beneficial services in socioeconomically disadvantaged populations.^24,25,26,27,28,29,30,31^

Scheduling barriers represent another multifaceted challenge. They may reflect challenges in patient outreach, such as variable work schedule flexibility or technology proficiency and access.^13,32,33^ Language barriers exacerbate these challenges.^34^ Enhanced language services, including interpreter-first outbound calls or expanded interpreter coverage, could mitigate scheduling disparities. Care coordination and addressing transportation challenges may be particularly critical for Medicare dual or disabled patients with complex care needs.^28,35^ Insurance status often serves as a proxy for broader socioeconomic inequities, driving both logistical barriers and clinician biases across all stages of follow-up care.

Variation in service completion primarily reflected the cascading effects of earlier-stage barriers. Although conditional completion rates after an order were high, they were insufficient to mitigate differences introduced at ordering and scheduling. Early barriers prevented many patients from progressing to later stages; possible examples include communication gaps during care episodes or at discharge, coordination challenges, or limited appointment availability. Potential interventions to overcome these early attrition points could include auto-scheduling with opt-out features, warm handoffs to care coordinators, or prearranged transportation support.

Temporal analyses revealed that early interventions are needed to address CAD testing variation, which emerged rapidly after index ED visits, while more sustained follow-up efforts are required for cardiology referrals. Our findings may also underestimate inequities arising at earlier access points given that the cohort was restricted to patients with established primary care, and thus, relatively advantaged via outpatient engagement at baseline.

A key contribution of this study is distinguishing between variation due to multiple intermediate stages vs a single, decisive bottleneck in a care cascade. When differences occur across several stages, resolving 1 difference cannot fully eliminate overall variation, due to downstream residual barriers. For example, Medicare dual-eligible or disabled patients were less likely to both receive a cardiology referral order and schedule the ordered visit; thus, improving ordering alone would still leave a substantial gap in completion. Conversely, when attrition is concentrated at a single stage, targeted intervention at that point could markedly improve equity. Non-English primary language and female patients showed relative attrition only at ordering; addressing this single barrier could align their completion rates with reference groups. Additionally, when differences accumulate across multiple steps, their effects multiply; each stage conditions the probability of success at the next, creating a cascading effect that requires broader interventions. Our study identified whether differences accumulated multiplicatively or arose from isolated bottlenecks, a distinction essential to designing effective, efficiently scoped interventions. By linking specific cascade stages to concrete interventions, health systems can pilot tailored approaches to close care gaps efficiently.

Findings also illustrate why standard quality metrics may understate true variation. Measures that condition on clinical orders exclude all patients who never received the order, obscuring potential root-causes of inequity. Examples include abnormal mammography follow-up measures and emerging colorectal cancer screening follow-up measures, both of which are dependent on an initial test order.^36,37^ Since conditioning on an order selects a group already receiving more engaged care, downstream metrics may overstate measured quality and underestimate or distort disparities. This potential bias underscores the importance of examining intermediate steps within care cascades.

ECG-derived risk score and sensitivity analyses indicated that differences persisted after accounting for residual cardiovascular risk. Adjustment for clinical severity would typically be expected to reduce observed differences if they were primarily driven by baseline clinical risk differences; however, results remained substantively robust, aligning with prior evidence of inequities among higher-risk patients. Fixed-effects analyses indicated that patient sorting, ED site, neighborhood (postal code), and temporal trends were insufficient to account for these differences, implicating point-of-care clinical decision-making or workflow processes as focal areas for improvement. While some variation in pretest probability of obstructive CAD may remain, adjustment for ECG-derived ischemia risk, a clinically relevant marker of short-term cardiac risk, enabled more equivalent comparisons across groups. Persistent variation after this adjustment suggests that differences in care delivery or follow-up access, rather than solely clinical appropriateness, drove the observed variation.

While this study used data from 1 health system, the stepwise framework introduced for dissecting care cascades is broadly generalizable. Health systems with diverse patient populations and challenges can leverage this approach to identify precise intervention points. Beyond health care, multistage analyses have illuminated how disparities accumulate and influence outcomes, such as how bias compounds across multiple stages of the criminal justice pretrial detention process.^38^ By isolating the precise stages where variation emerges, this framework offers a structured tool to strategically allocate resources and tailor interventions to drive meaningful equity improvements across diverse systems.

### Limitations

This study used EHR data from a single academic health system, which may limit generalizability. However, the health system encompasses several large hospitals and clinics across multiple states, serving a diverse patient population. Our primary aim was to develop a stepwise framework adaptable by other health systems to identify drivers of variation within their own populations. While focused on cardiovascular follow-up due to its clinical burden and prevalence, this framework is applicable to other conditions. As with all observational research, unmeasured clinical risk factors may exist; adjustments using a validated ECG-derived cardiovascular risk score and sensitivity analyses intended to mitigate this. Additional limitations include potential measurement bias from care received outside the system and incomplete capture of ED presentation details that may influence follow-up decisions. Although practical, conditioning on intermediate steps may introduce selection bias that limits full causal interpretation. The exploratory nature of this work may increase the potential for false positives; this study was intentionally structured to generate insights for future investigations and targeted interventions. Given variation across health systems in demographic characteristics and structural factors, results may not directly generalize, highlighting the need for locally tailored analyses. Despite these limitations, the use of granular, time-stamped EHR metadata enabled application of a novel, stepwise care cascade framework to identify actionable points to advance equity in health care.

## Conclusions

This cohort study identified substantial variation in cardiovascular follow-up care after ED visits, concentrated primarily in the early ordering and scheduling stages of care cascades. Differences were most pronounced among patients with noncommercial insurance, with additional variation by primary language and sex; differences persisted after adjustment for clinical characteristics, including ECG-derived cardiovascular risk. By mapping differences across discrete cascade stages, including ordering, scheduling, and completion, this work introduces a novel, reproducible framework to identify where and for whom inequities arise. Applying similar stepwise analyses across health systems can guide targeted, stage-specific interventions. Broader adoption of this approach offers an actionable pathway to close persistent care gaps and improve equity and efficiency in health care delivery.

## Notes

Supplement 1. eMethods. Data Details, Study Design, and Implementation Framework eFigure 1. Study Cohort Flow Chart eFigure 2. Simplified Study Schema eFigure 3. Sensitivity Analyses for Follow-Up Care Cascade Completion After Emergency Department (ED) Visits, Stratified by Race or Ethnicity eTable 1. Adjusted Odds Ratios for Completion of Cardiovascular Follow-Up Care Endpoints at Varying Time Points After an Index Emergency Department (ED) Visit eTable 2. Adjusted Probabilities of Completion of Cardiovascular Follow-Up Care Endpoints and Intermediate Steps eTable 3. Bounding Analyses Using E-values eTable 4. Adjusted Odds Ratios for Referral Order by Subgroup Using IPCW-Weighted and Unweighted Logistic Regression Models eTable 5. Emergency Department (ED) Site-Level Heterogeneity via Random Intercept Mixed-Effects Models eTable 6. Time to Completion of Cascade Steps eTable 7. Adjusted Completion Rates of Primary Care Office Visits within Six Months After an Index ED Visit eTable 8. Unadjusted Completion Rates of Procedures for Coronary Artery Disease within Six Months After an Index ED Visit eTable 9. Adjusted Completion Rates of Procedures for Coronary Artery Disease within Six Months After an Index ED Visit

Supplement 2. Data Sharing Statement
