# Supplementary material for: Sources of Variation in Cardiovascular Care Cascades
Source: JAMA Health Forum. 2026 Apr 3;7(4):e260491. doi: 10.1001/jamahealthforum.2026.0491 (PMC13049495; doi:10.1001/jamahealthforum.2026.0491)
Supplement: Supplement 1. — eMethods. Data Details, Study Design, and Implementation Framework eFigure 1. Study Cohort Flow Chart eFigure 2. Simplified Study Schema eFigure 3. Sensitivity Analyses for Follow-Up Care Cascade Completion After Emergency Department (ED) Visits, Stratified by Race or Ethnicity eTable 1. Adjusted Odds Ratios for Completion of Cardiovascular Follow-Up Care Endpoints at Varying Time Points After an Index Emergency Department (ED) Visit eTable 2. Adjusted Probabilities of Completion of Cardiovascular Follow-Up Care Endpoints and Intermediate Steps eTable 3. Bounding Analyses Using E-values eTable 4. Adjusted Odds Ratios for Referral Order by Subgroup Using IPCW-Weighted and Unweighted Logistic Regression Models eTable 5. Emergency Department (ED) Site-Level Heterogeneity via Random Intercept Mixed-Effects Models eTable 6. Time to Completion of Cascade Steps eTable 7. Adjusted Completion Rates of Primary Care Office Visits within Six Months After an Index ED Visit eTable 8. Unadjusted Completion Rates of Procedures for Coronary Artery Disease within Six Months After an Index ED Visit eTable 9. Adjusted Completion Rates of Procedures for Coronary Artery Disease within Six Months After an Index ED Visit [file jamahealthforum-e260491-s001.pdf]

Supplemental Online Content

Wang AZ, Shanmugam D, Divakaran S, Pierson E, Barnett ML. Sources of variation in cardiovascular care cascades. *JAMA Health Forum.* Published online April 3, 2026. doi:10.1001/jamahealthforum.2026.0491

eMethods. Data Details, Study Design, and Implementation Framework

eFigure 1. Study Cohort Flow Chart

eFigure 2. Simplified Study Schema

eFigure 3. Sensitivity Analyses for Follow-Up Care Cascade Completion After Emergency Department (ED) Visits, Stratified by Race or Ethnicity

eTable 1. Adjusted Odds Ratios for Completion of Cardiovascular Follow-Up Care Endpoints at Varying Time Points After an Index Emergency Department (ED) Visit

eTable 2. Adjusted Probabilities of Completion of Cardiovascular Follow-Up Care Endpoints and Intermediate Steps

eTable 3. Bounding Analyses Using E-values

eTable 4. Adjusted Odds Ratios for Referral Order by Subgroup Using IPCW-Weighted and Unweighted Logistic Regression Models

eTable 5. Emergency Department (ED) Site-Level Heterogeneity via Random Intercept Mixed-Effects Models

eTable 6. Time to Completion of Cascade Steps

eTable 7. Adjusted Completion Rates of Primary Care Office Visits within Six Months After an Index ED Visit

eTable 8. Unadjusted Completion Rates of Procedures for Coronary Artery Disease within Six Months After an Index ED Visit

eTable 9. Adjusted Completion Rates of Procedures for Coronary Artery Disease within Six Months After an Index ED Visit

This supplemental material has been provided by the authors to give readers additional information about their work.

© 2026 Wang AZ et al. *JAMA Health Forum*.

eMethods: Data Details and Study Design

A. Data Sources

Electronic health record (EHR) data for this study were obtained from an Electronic Data Warehouse (EDW), which consolidates clinical, financial, and operational data from the complete network of hospitals and affiliated clinics within the health care system studied. The EDW serves as a centralized repository, consolidating a large volume of data elements from multiple source systems, and provides detailed information on patient encounters, diagnoses, procedures, medications, and test results, all organized with structured metadata. Data extraction was performed using SQL queries based on predefined inclusion criteria.

Data included all patients with Emergency Department (ED) visits with a linked troponin order from 2016 to 2022. To ensure a patient-level analysis, the earliest ED visit for each patient during the study period was considered. The analysitic sample was restricted to patients aged 18 years or older at the time of the index ED visit.

Inclusion criteria required patients to have at least one encounter recorded with a primary care department within the two years prior to their ED visit. Patients who had any completed cardiology referral visit step (order, scheduling, or completed visit time stamps) within one year prior to their ED visit were excluded, as were patients with any completed coronary artery disease (CAD) testing step (order, scheduling, or completed test time stamps) during the same period. Additionally, patients with a prior ICD-10 coded diagnosis of ischemic heart disease were excluded. To ensure data completeness, only patients with complete demographic data were included, resulting in the exclusion of 5.3% of patients. Collectively, these criteria were intended to identify patients receiving care through the health system, yet with the least diagnostic cardiovascular testing already performed.

Definitions of cardiology referral and CAD testing are included in “Outcome Variables” below. Further details on primary care departments and ICD-10 codes for ischemic heart disease are included in “Inclusion and Exclusion Criteria Variables” below. A study cohort flow chart is included in eFigure 1.

B. Health System Context

Payer Mix
As of 2022, the health system’s patient population was covered primarily by commercial insurance (57%), followed by Medicare (22%), Medicare Advantage (8%), Medicaid (8%), and other payers (5%).

Interpreter Context
The health system provides free interpreter services for patients whose primary language is not English, who have limited English proficiency, or who are Deaf or hard of hearing. In-person interpreters are available for the most common languages during

© 2026 Wang AZ et al. *JAMA Health Forum*.

core working hours, and phone or video interpreters are commonly used both during and outside of working hours for other languages or when in-person interpreters are not available.

C. Outcome Measures

Cardiology Referral Visits
All cardiology referral orders, scheduling time stamps, and cardiology visits occurring during the study period were extracted. Cardiology referral orders were defined as outpatient referral orders for consultation with cardiology as the requested specialty. Cardiology referral visits included “office visit” or “appointment” encounter types within a cardiology department. The dataset was reduced to patient-level by selecting the earliest completion date for each intermediate step (order, scheduling, or visit completion) following the ED visit for each patient.

Coronary Artery Disease (CAD) Testing: Cardiac Stress Testing and Coronary Computed Tomography Angiography (CTA)
CAD testing collectively refers to stress testing or coronary CTA. All orders, scheduling time stamps, and completion of CAD testing occurring during the study period were extracted. CAD tests included in the analysis were categorized using the EHR test labels as follows:

Test Labels for Coronary Artery Disease (CAD) Testing

Test Type	Test Label in EHR
Exercise Stress Test (ECG)	Stress Test Exercise
Nuclear Stress Test	NC Myocardial Perfusion Exercise, Multi; NC Myocardial Perfusion Pharmacologic Stress Multiple; NC100 (Tech Order Only) NC Stress Test with Nuclear Imaging; NC Myocardial Perfusion Stress Single; MIBI Stress Test; Nuclear Stress Test; Stress Test Pharmacologic; NC PET/CT Cardiac Perfusion Multiple; NC PET Cardiac Perfusion Single
Stress Echocardiogram	Stress Echo Exercise; Stress Echo Dobutamine
Cardiac MRI Stress Imaging	MRI Cardiac Stress
Cardiac Computed Tomography Angiography (CTA)	CT Angio Cardiac Structure and Morphology with Contrast; CT Angio Coronary Arteries

The dataset was reduced to patient-level by selecting the earliest completion date for any intermediate step (order, scheduling, or test completion) following the ED visit for each patient.

D. Inclusion and Exclusion Criteria Variables

A qualifying primary care visit for study inclusion was defined as an appointment or office visit within one of the following specialty departments: Internal Medicine, Family Medicine, Gerontology, or Primary Care.

Patients with any diagnoses of ischemic heart disease prior to their Emergency Department (ED) visits were excluded, as determined by the ICD-10 codes as follows:

Diagnosis Codes for Ischemic Heart Disease

ICD-10 Code	Description
**I20**	**Angina pectoris**
I20.0, I20.1, I20.8, I20.9	Unstable angina; Angina pectoris with documented spasm; Other forms of angina pectoris; Angina pectoris, unspecified
**I21**	**Acute myocardial infarction**
I21.0, I21.1, I21.2, I21.3, I21.4, I21.9	ST elevation (STEMI) myocardial infarction of anterior wall; ST elevation (STEMI) myocardial infarction of inferior wall; ST elevation (STEMI) myocardial infarction of other sites; ST elevation (STEMI) myocardial infarction of unspecified site; Non-ST elevation (NSTEMI) myocardial infarction; Acute myocardial infarction, unspecified
**I22**	**Subsequent ST elevation (STEMI) and non-ST elevation (NSTEMI) myocardial infarction**
I22.0, I22.1, I22.2, I22.8, I22.9	Subsequent ST elevation (STEMI) myocardial infarction of anterior wall; Subsequent ST elevation (STEMI) myocardial infarction of inferior wall; Subsequent non-ST elevation (NSTEMI) myocardial infarction; Subsequent ST elevation (STEMI) myocardial infarction of other sites; Subsequent ST elevation (STEMI) myocardial infarction of unspecified site
**I23**	**Certain current complications following ST elevation (STEMI) and non-ST elevation (NSTEMI) myocardial infarction (within the 28-day period)**
I23.0, I23.1, I23.2, I23.3, I23.4, I23.5, I23.6, I23.7, I23.8	Hemopericardium as current complication following acute myocardial infarction; Atrial septal defect as current complication following acute myocardial infarction; Ventricular septal defect as current complication following acute myocardial infarction; Rupture of cardiac wall without hemopericardium as current complication following acute myocardial infarction; Rupture of chordae tendineae as current complication following acute myocardial infarction; Rupture of papillary muscle as current complication following acute myocardial infarction; Thrombosis of atrium, auricular appendage, and ventricle as current complications following acute myocardial infarction; Postinfarction angina; Other current complications following acute myocardial infarction
**I24**	**Other acute ischemic heart diseases**
I24.0, I24.1, I24.8, I24.9	Acute coronary thrombosis not resulting in myocardial infarction; Dressler's syndrome; Other forms of acute ischemic heart disease; Acute ischemic heart disease, unspecified
**I25**	**Chronic ischemic heart disease**
I25.1, I25.2, I25.3, I25.4, I25.5, I25.6, I25.7, I25.8, I25.9	Atherosclerotic heart disease of native coronary artery; Old myocardial infarction; Aneurysm of heart; Coronary artery aneurysm and dissection; Ischemic cardiomyopathy; Silent myocardial ischemia; Atherosclerosis of coronary artery bypass graft(s) and coronary artery of transplanted heart with angina pectoris; Other forms of chronic ischemic heart disease; Chronic ischemic heart disease, unspecified

© 2026 Wang AZ et al. *JAMA Health Forum*.

For the CAD testing analyses, patients receiving invasive coronary angiography during the index episode were excluded, as the study focused on lower- to medium-acuity presentations where discretionary use of non-invasive testing may contribute to disparities. The relevant procedure labels in the EHR included: Percutaneous Coronary Intervention (PCI), Coronary Arteriogram with Possible Intervention, Coronary Arteriogram, Left Heart Catheterization, or Right and Left Heart Catheterization.

E. Study Variables

1) Emergency Department Visit

Inpatient Status
Disposition after the ED visit was categorized as either inpatient admission or discharge. Patients routed to an ED observation unit were categorized based on their subsequent disposition status: inpatient admission if the encounter included an inpatient admission time stamp, discharge otherwise.

Troponin Orders and Results
Troponin levels were extracted as the highest available troponin level lab result linked to the index ED visit and resulting between ED arrival and 72 hours post-ED departure, regardless of disposition. Abnormal or elevated troponin was defined as a troponin level greater than the upper limit of normal, as determined by the reference level specific to the troponin test used. In regression models, magnitude of troponin elevation was represented by categories of a) within normal limits, b) one to three times the upper limit of normal, and c) greater than three times the upper limit of normal, in alignment with its use in the HEART risk stratification score.[1–3]

2) Patient Characteristics

Demographic information, including sex, primary language, and date of birth, was obtained from the patient characteristics file in the EHR.

Age
Age was calculated at the time of the ED visit arrival using each patient's date of birth.

Comorbidities
Comorbidities were determined using ICD-10 codes linked to billed EHR encounters in the year prior to the ED visit. The count of distinct ICD-10 codes per patient was recorded as the total number of comorbidities.

Insurance Type
Insurance type was defined based on each patient's primary effective insurance payer at the time of the index ED visit. Insurance types were categorized as follows: Medicaid (including Medicaid, Health Safety Net, MassHealth, Free Care, ConnectorCare, and Neighborhood Health Plan), Medicare, Medicare-Disabled (as indicated in the patient

© 2026 Wang AZ et al. *JAMA Health Forum*.

insurance record), and Commercial (any active commercial insurance plan). Patients enrolled in both Medicare and Medicaid were classified as Medicare-Dual. Other insurance types included Other Government Programs, Workers' Compensation, Motor Vehicle, or International. Patients with primary insurance within the Other insurance category were excluded from analyses, representing N=114 patients. Medicare Advantage patients were included within non-commercial insurance groupings based on their specific plan labels, and in exploratory analyses, exhibited endpoint completion patterns similar to those of patients with non-commercial insurance types with respect to similar completion barriers.

Race/Ethnicity Classification
Race and ethnicity data were obtained from self-reported patient race/ethnicity in the patient characteristics file in the EHR. Patients were first categorized as either Hispanic or Latino, or Not Hispanic or Latino, then further classified by specific race within those categories. The recorded race categories included: American Indian or Alaska Native, Asian, Black or African American, Declined, Hispanic or Latino, Native Hawaiian or Other Pacific Islander, Other, Unavailable, and White.

Patients were grouped into the following race/ethnicity subgroups: Black, Non-Hispanic; Hispanic or Latino; White, Non-Hispanic; and Asian and All Other, Non-Hispanic. Due to low patient numbers, specific race/ethnicity groups within the Asian and All Other, Non-Hispanic category were combined.

In cases where multiple race indicators were present for a single patient, the most specific race category was used according to the following predefined order: American Indian or Alaska Native, Native Hawaiian or Other Pacific Islander, Black or African American, Hispanic or Latino, Asian, White, Declined, Unavailable, and Other. This approach ensured that the most specific race indicator was selected to reflect potential societal and structural factors that could influence health outcomes; these factors are not always captured by broader or default race categories (e.g., Unavailable or White). Notably, this further specification affected a limited number of patient entries at 1.3%.

F. ECG-Derived Cardiovascular Risk Score

Electrocardiogram (ECG)-Derived Cardiovascular Risk Score
We developed an ECG-derived cardiovascular risk score using a dilated convolutional neural network (CNN), fine-tuned from the PreOpNet architecture.[4] The model predicts the likelihood of abnormal cardiac perfusion, as confirmed by nuclear stress tests. Inputs included ECG waveform data and demographic features (age, race, and sex).

Model Training, Development, and Validation
The model was trained using data from patients who underwent nuclear stress tests between 2016 and 2019. Exclusions included inconclusive test results, exercise stress tests (due to higher variability), and patients with incomplete demographic data. The final dataset comprised 14,561 ECG-stress test pairings from 14,561 patients, where we selected the most recent ECG for each patient (ensuring that the ECG is not performed

on the same day as the stress test). We selected a single ECG per patient to avoid bias from patients with multiple ECGs. ECGs were required to occur within 6 months prior to the stress test. The CNN was optimized to predict positive stress tests (evidence of abnormal cardiac perfusion) using cross-entropy loss. We then recalibrated the model by fitting a logistic regression to predict the likelihood of a positive stress test given the risk score, age, race, and sex (using a held-out set of recalibration examples). We allocated 10,920 examples for model training, 1,092 examples for model recalibration, and 1,121 examples for model validation.

Imaging-based stress test results (e.g., myocardial perfusion imaging, PET/CT cardiac perfusion) served as the primary reference standard. Performance metrics, including the area under the receiver operating characteristic curve (AUC), were calculated to assess model discrimination. The trained model achieved an AUC of 0.76 on a held-out set of patients, outperforming a logistic regression applied to demographic features (AUC of 0.60) and a logistic regression applied to preprocessed ECG features (AUC of 0.66) by a large margin.

Calibration analysis showed that predicted risk scores closely aligned with observed stress test outcomes across different risk strata. Subgroup analyses demonstrated consistent model performance across race, insurance, and sex subgroups. Specifically, using the Spiegelhalter test, we fail to reject the null hypothesis of model miscalibration across race, insurance, and sex subgroups.

Electrocardiogram (ECG)-Derived Cardiovascular Risk Score Model Performance Compared to Simpler Models

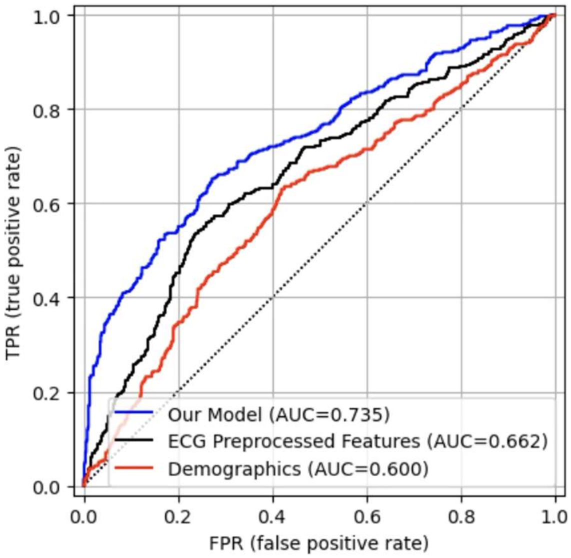

To assess transportability of the ECG-derived risk score to the study cohort (patients with troponin testing), we conducted a robustness analysis in which the held-out validation sample was reweighted to match the study cohort's age, race/ethnicity, sex, and hospital site distributions. This analysis evaluates sensitivity of model performance

© 2026 Wang AZ et al. *JAMA Health Forum*.

to demographic differences between cohorts and is consistent with established methods for accounting for covariate shift.[5,6] Subgroup discrimination and calibration before and after reweighting are shown in tables below.

Reweighting resulted in minimal changes in calibration error and AUC across insurance type, race/ethnicity, and primary language subgroups. Calibration error was unchanged or modestly improved after reweighting, and discrimination remained similar to the original validation estimates. Stable model performance after reweighting suggests minimal residual miscalibration attributable to demographic differences between cohorts, consistent with transportability of the risk score to the troponin-tested study population.

Performance of Original and Reweighted Models Across Patient Subgroups: Model Calibration and Discrimination

Insurance Type	Reweighted Calibration Error	Original Calibration Error	Reweighted AUC	Original AUC
Commercial	0.06	0.08	0.75	0.71
Medicaid	0.08	0.05	0.77	0.79
Medicare	0.09	0.09	0.70	0.70

Race/Ethnicity	Reweighted Calibration Error	Original Calibration Error	Reweighted AUC	Original AUC
Black, Non-Hispanic	0.05	0.05	0.69	0.71
Hispanic or Latino	0.06	0.07	0.72	0.72
White, Non-Hispanic	0.05	0.06	0.73	0.73

Primary Language	Reweighted Calibration Error	Original Calibration Error	Reweighted AUC	Original AUC
English	0.09	0.09	0.70	0.72
Spanish	0.08	0.10	0.71	0.69

© 2026 Wang AZ et al. *JAMA Health Forum*.

Demographic and Clinical Characteristics of the Development and Study Cohorts

Patient Group	Development Cohort	Study Cohort
Race: White, Non-Hispanic (%)	75.3	71.6
Race: Black, Non-Hispanic (%)	9.2	9.7
Race: Hispanic or Latino (%)	7.1	10.9
Race: Asian (%)	3.4	3.5
Race: Declined or Unavailable (%)	3.2	2.5
Race: Other (%)	1.7	1.5
Race: Native American or Pacific Islander (%)	0.2	0.3
Male (%)	56.2	42.4
Site 1 (%)	54.0	36.3
Site 2 (%)	44.2	33.0
Site 3 (%)	1.4	12.7
Site 4 (%)	0.3	0.1
Site 5 (%)	0.1	1.0
Site 6 (%)	0.1	0.0
Site 7 (%)	0.0	16.9
Age (mean [IQR])	67.9 [60.0, 76.4]	61.1 [48.6, 74.4]

Study Sample
The model was applied to a cohort of patients presenting to the emergency department (ED) with troponin orders between 2020 and 2022. Inclusion criteria required at least one ECG performed on or before the ED visit, no stress tests, cardiology referrals, or cardiology visits in the preceding year, and at least one primary care visit within the two years prior to the ED visit. After applying these filters, 32,951 patients were assigned ECG-derived cardiovascular risk scores using the model.

The final study sample include patients with ECG-derived cardiovascular risk scores above the sample median. In a validation sample, patients above the sample median threshold of risk score were 2.8-fold more likely to have abnormal cardiac perfusion on stress test imaging compared with those below the median (29.6% abnormal perfusion rate within 6 months across all patients).

© 2026 Wang AZ et al. *JAMA Health Forum*.

G. Sensitivity and Supplemental Analyses

Primary and Exploratory Analyses
We performed a range of subgroup and sensitivity analyses. Primary pre-specified contrasts included adjusted comparisons by insurance type, race/ethnicity, primary language, and sex at key cascade steps (order, conditional scheduling, conditional completion, unconditional completion). A simplified study schema representing the main analyses is included in eFigure 2. Additional analyses, such as those excluding patients with reduced kidney function, restricted based on troponin level or HEART score, additional fixed effects and random effects analyses, and using varying ECG risk thresholds, are considered exploratory or hypothesis-generating. Specifications for additional analyses are detailed below.

Supplemental Analyses: Temporal Analyses (eTable 1)
To assess whether and how cardiovascular care completion evolved over time, we conducted analyses replicating the primary endpoint analyses at varying time points after the index ED visit.

1. Analysis of endpoint completion using 15, 30, 45, and 180 days as the time point of interest after the index ED visit.

Supplemental Analyses: Adjusted Probabilities (eTable 2)

2. Calculated adjusted predicted probabilities and risk differences by subgroup for each cascade step and overall completion, corresponding to the main analyses.

Sensitivity Analyses (eFigure 2)

We conducted an analysis excluding patients with reduced kidney function (estimated Glomerular Filtration Rate (eGFR) < 60 mL/min/1.73 m^2), as these patients may have elevated troponin levels without coronary disease. eGFR values were extracted from all laboratory results per patient in the year prior to and including the ED visit.

3. Exclusion of patients with reduced kidney function, defined by eGFR < 60 mL/min/1.73 m^2 anytime in the year prior to and including the index ED visit date.

To examine whether observed variations persisted among higher cardiovascular risk groups, we performed subgroup analyses of endpoint outcome measures for cardiology referral visits and CAD testing:

4. Restriction to individuals with elevated troponin levels, defined as values exceeding the laboratory reference range upper limit.
5. Restriction to individuals aged 65 years or older within the highest troponin level category. Given their age and highly elevated troponin (>3x upper limit), these individuals qualify as intermediate risk or higher under ACC/AHA chest pain

© 2026 Wang AZ et al. *JAMA Health Forum*.

guidelines using the HEART score and are indicated for specific diagnostic imaging (stress testing/coronary CTA or coronary angiography) or intervention.[1–3]

To evaluate other potential contributing or confounding factors in the analysis, including site-specific, temporal, or unobserved clinical risk factors, additional analyses included:

6. Emergency department fixed effects (indicator variables) to account for unmeasured site-level factors that could influence referral or follow-up patterns.
7. Month-year fixed effects to account for temporal changes in clinical practice or referral decisions.
8. Patient ZIP code fixed effects covariate as a neighborhood-level SES to capture unmeasured socioeconomic factors.

To assess robustness to the ECG risk score threshold used:

9. Sensitivity analyses applying alternative ECG risk score cutoffs, including upper tertile and upper quartile thresholds, compared to the median threshold used in the main analysis.

Supplemental Analyses: Bounding Analyses Using E-values (eTable 3)

10. To evaluate the potential impact of unmeasured confounding on adjusted associations, we calculated E-values for key covariates (insurance type, race/ethnicity, language, sex) across primary outcomes related to CAD testing and cardiology referral visits.

E-values represent the minimum strength of association an unmeasured confounder would need with both the exposure and outcome to fully explain the observed effect.[7] For example, an E-value of 2.24 for Medicare Dual or Disabled in the Received Test Order outcome indicates that a confounder associated with both exposure and outcome by at least this magnitude could negate the observed association. Larger E-values indicate greater robustness to unmeasured confounding. We calculated E-values for results with 95% confidence intervals excluding the null (OR = 1).

Sensitivity Analyses: Inverse Probability of Censoring Weighting (IPCW) and Unweighted Logistic Regression (eTable 4)

To evaluate for potential bias from informative censoring due to death before six months of follow-up, we conducted sensitivity analyses using inverse probability of censoring weighting (IPCW). We modeled the hazard of death censoring with a Cox proportional hazards model including the same baseline covariates as in the primary analyses. From this, we derived stabilized IPC weights reflecting each individual's probability of remaining uncensored through their observed follow-up time. The main logistic regression models for each referral and CAD testing step were refit, incorporating these IPCW weights to adjust for potential informative censoring. IPCW-adjusted estimates were compared to primary unweighted models to assess robustness.

© 2026 Wang AZ et al. *JAMA Health Forum*.

11. Sensitivity analyses comparing inverse probability of censoring weighted (IPCW) logistic regression to standard unweighted adjusted logistic regression to assess the robustness of our findings.

Supplemental Analyses: ED Site-Level Heterogeneity via Random Intercept Mixed-Effects Models (eTable 5)

12. Assessment of variability in referral and follow-up patterns across ED sites using random intercept mixed-effects models to account for unmeasured site-level factors.

Supplemental Analyses: Time to Completion of Cascade Steps (eTable 6)

To evaluate whether differences in care completion were accompanied by differences in time to care among those who ultimately received testing or referral, we examined time from the index ED visit to completion of CAD testing and cardiology referral visits.

13. Analysis of days from the index ED visit to completion of outcome (CAD testing or cardiology referral visits), summarized by patient demographic and clinical subgroups.

Supplemental Analyses: Primary Care Office Visits (eTable 7)

We evaluated completion of office visits in a primary care department within six months after the index ED visit.

14. Analysis of completion of primary care visits within six months after the index ED visit.

Supplemental Analyses: Procedure Completion (eTables 8 and 9)

We evaluated completion of invasive coronary angiography (ICA) or coronary artery bypass surgery (CABG), two procedures for coronary artery disease, within six months after the index ED visit.

15. Analysis of completion of ICA within six months after the index ED visit.
16. Analysis of completion of CABG within six months after the index ED visit.

For the Procedure Completion Supplemental Analyses, all coronary artery bypass graft (CABG) surgery or invasive coronary angiography (ICA) EHR entries occurring during the study period were extracted. CAD procedures in the analysis were identified using the procedure labels below. The dataset was reduced to patient-level by selecting the earliest CAD procedure occurring on or after each patient’s ED arrival date per patient.

© 2026 Wang AZ et al. *JAMA Health Forum*.

Procedure Labels for Procedures for Coronary Artery Disease (CAD)

Procedure Label in EHR
Coronary Artery Bypass Graft Angiogram with Possible Intervention
Coronary Artery Bypass Graft Angiogram
Coronary Artery Bypass Graft
Coronary Arteriogram with Possible Intervention
Coronary Arteriogram
Percutaneous Coronary Intervention (PCI)
Left Heart Catheterization
Right and Left Heart Catheterization

H. Implementation Framework for Order-Schedule-Complete Cascade Logic and Analysis

This implementation framework outlines key steps to extract and analyze order, scheduling, and completion events from EHR data to apply the cascade framework to other care outcomes or health systems.

1. **Define Study Population:**

   Extract relevant visits within a set timeframe. Apply inclusion/exclusion criteria (e.g., adult patients, visit includes a relevant clinical order).
   Example: For this study, we identified Emergency Department visits with an associated troponin order, followed by patient-level exclusion criteria.

2. **Identify Clinical Orders for the Outcome of Interest:**
   Select relevant orders using local codes.

   Examples: See "Test Labels for Coronary Artery Disease Testing" or "Cardiology Referral Visits" as defined above.

3. **Match Orders to Scheduling:**

   Use unique order IDs within the EHR to link each order to its corresponding scheduling record.

   Identify dates of scheduled visits/tests, accordingly, if present.

4. **Confirm Completion:**

   Identify dates of completion to verify whether services, visits, or procedures were completed after scheduling.

5. **Aggregate at Patient Level:**

   Consolidate earliest valid order, scheduling instance, and completion events per patient, preserving the chronological sequence of events.

© 2026 Wang AZ et al. *JAMA Health Forum*.

6. **Add Patient Covariates:**
   Link demographics, insurance payer, comorbidities, and other relevant factors at the patient level using EHR data.
7. **Analyze Cascade Progression:**

   Calculate proportions completing each step by patient subgroup to identify unadjusted differences. Then, use step-wise adjusted regression models to assess factors associated with progression through each cascade step:

   > **Step 1:** Completion of the initial required event (e.g., order placed).
   >
   > **Step 2 to Step X:** Progression to intermediate events (e.g., appointment scheduled), conditional on completion of the prior step. Multiple intermediate steps can be modeled as needed.
   >
   > **Step X+1:** Progression to the final event (e.g., service completed), conditional on the preceding step, Step X.

   Additionally, run unconditional models of overall completion to assess broad patterns across the full population.

© 2026 Wang AZ et al. *JAMA Health Forum*.

eFigure 1. Study Cohort Flow Chart

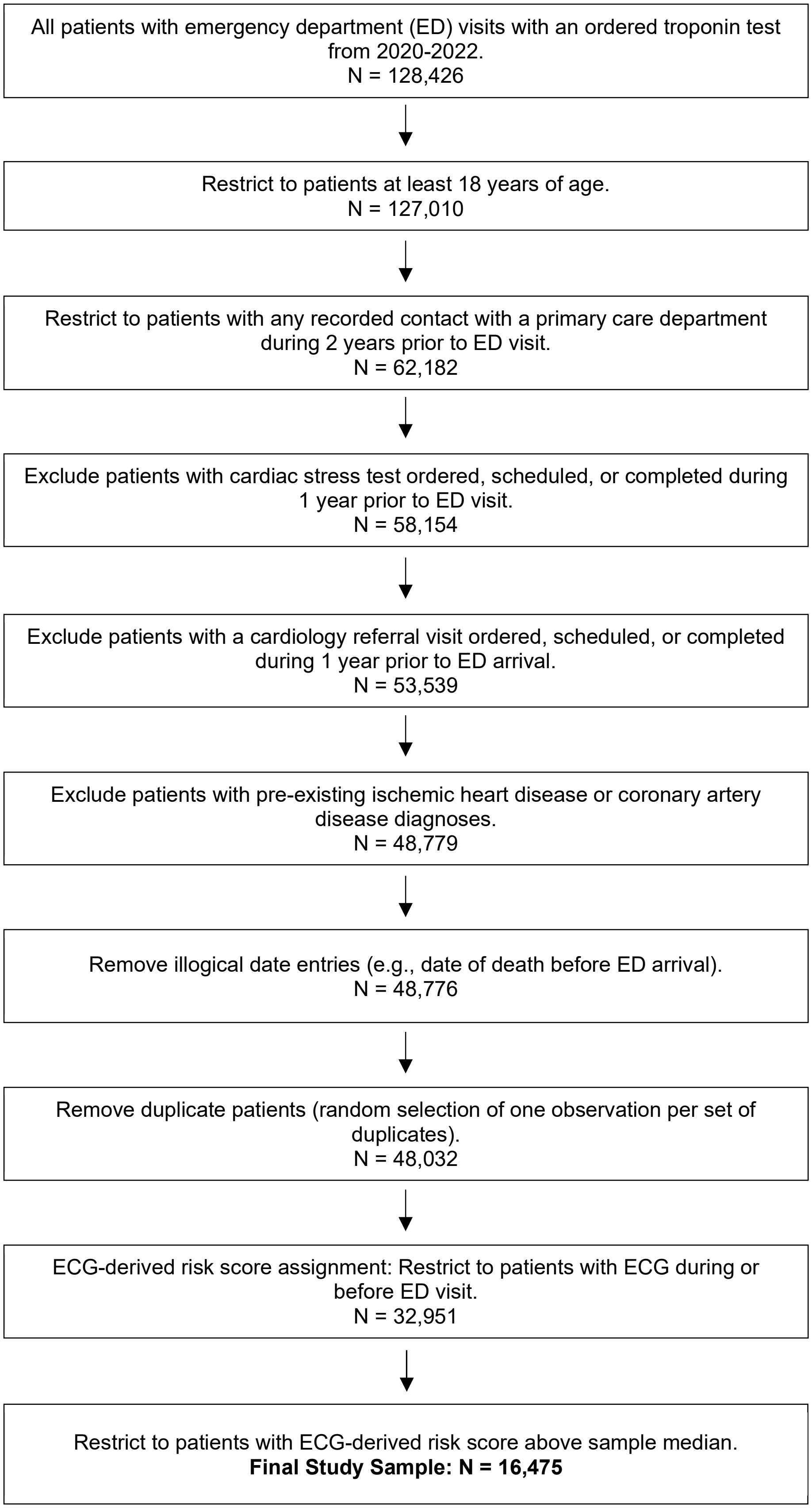

© 2026 Wang AZ et al. *JAMA Health Forum*.

eFigure 2. Simplified Study Schema

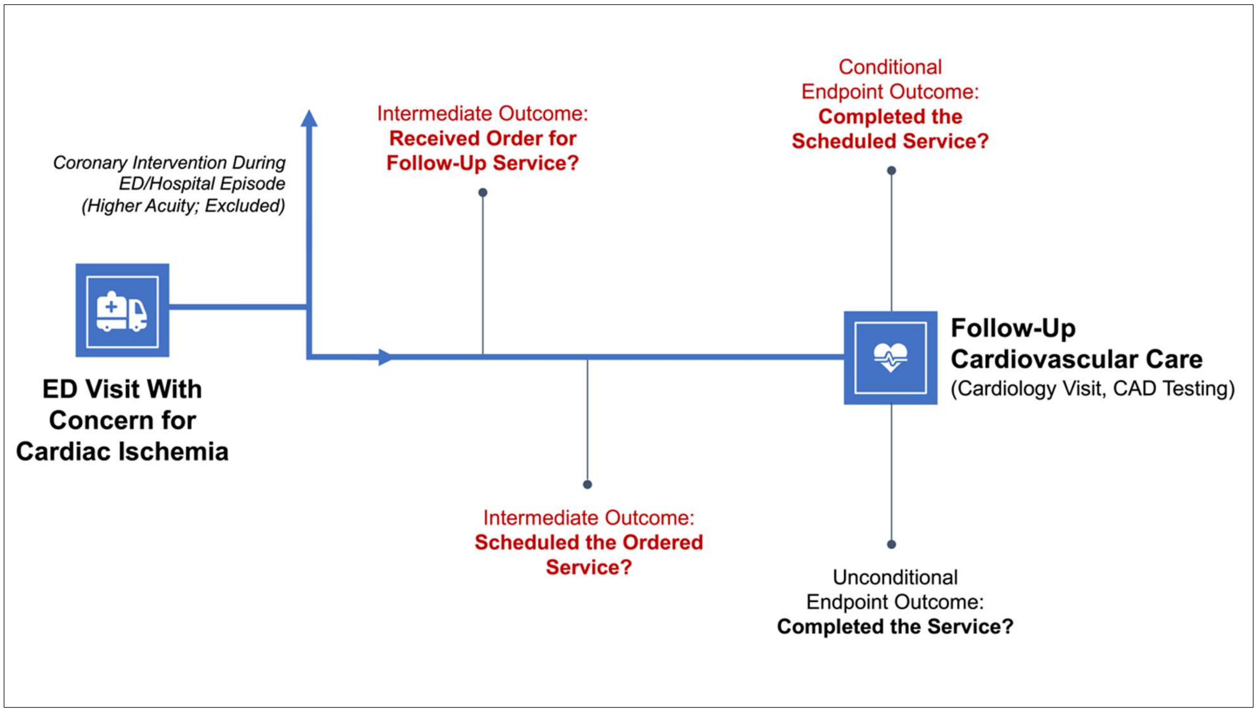

eFigure 2 Legend. Simplified study schema representing the main analyses of completion of key intermediate and endpoint steps within follow-up cardiovascular care cascades within six months of each patient's initial ED visit. CAD testing collectively refers to cardiac stress testing or coronary computed tomography angiography (CCTA). Abbreviations: ED, Emergency Department; CAD, Coronary Artery Disease.

© 2026 Wang AZ et al. *JAMA Health Forum*.

eFigure 3. Sensitivity Analyses for Cardiovascular Referral Cascade Completion After Emergency Department (ED) Visit

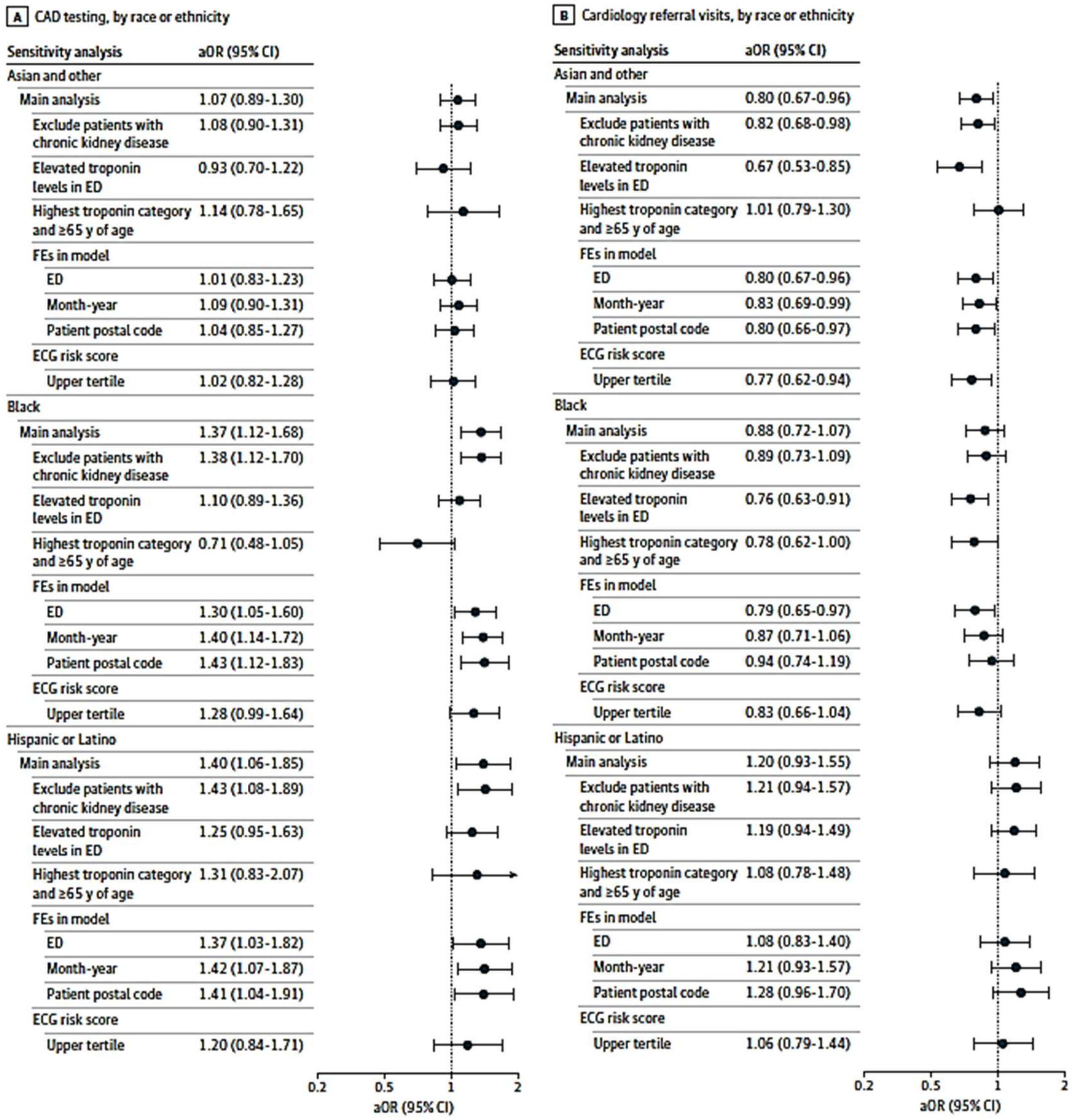

Models were adjusted for electrocardiogram-derived cardiovascular risk, age, sex, comorbidities, troponin magnitude, and admission status. For patients aged 65 years or older with troponin levels in the highest category included 110 Medicaid-insured patients due to near-universal additional Medicare coverage in this age group; this small sample likely contributed to the wider 95% CIs for this subgroup. Regarding race and ethnicity, these data were obtained from the electronic health record and reflect patient self-identification at registration; if not included in Hispanic/Latino, patient was not of Hispanic/Latino ethnicity; and Asian and other includes American Indian or Alaska Native, Asian, Native Hawaiian or Other Pacific Islander, other, or declined. aOR indicates adjusted odds ratio; ECG, electrocardiogram; FE, fixed effects.

© 2026 Wang AZ et al. *JAMA Health Forum*.

eTable 1. Temporal Trends: Adjusted Completion Rates of Cardiovascular Follow-Up Endpoints at Varying Time Points After an Index ED Visit

		Days after ED Visit							
		Completion of Coronary Artery Disease (CAD) Testing				Completion of Cardiology Referral Visit			
**Subgroup**	No. of Patients N=16,475	**15 Days**	**30 Days**	**45 Days**	**180 Days (Main Analysis)**	**15 Days**	**30 Days**	**45 Days**	**180 Days (Main Analysis)**
**Insurance Payer**									
Commercial	5,299	Ref	Ref	Ref	Ref	Ref	Ref	Ref	Ref
Medicare	7,480	0.62 (0.52, 0.74)	0.64 (0.54, 0.76)	0.64 (0.55, 0.76)	0.66 (0.57, 0.77)	1.06 (0.78, 1.43)	0.90 (0.74, 1.10)	0.88 (0.74, 1.05)	0.88 (0.78, 1.00)
Medicaid	1,759	0.76 (0.62, 0.95)	0.74 (0.61, 0.91)	0.71 (0.58, 0.86)	0.74 (0.62, 0.89)	0.69 (0.44, 1.07)	0.64 (0.48, 0.85)	0.67 (0.53, 0.85)	0.66 (0.55, 0.79)
Medicare Dual or Disabled	1,773	0.41 (0.32, 0.54)	0.41 (0.32, 0.52)	0.40 (0.31, 0.50)	0.45 (0.36, 0.56)	0.43 (0.27, 0.70)	0.46 (0.34, 0.62)	0.44 (0.35, 0.57)	0.47 (0.39, 0.57)
**Race/Ethnicity**									
Asian, Non-Hispanic	1,449	1.12 (0.90, 1.41)	**1.26 (1.03, 1.54)**	1.20 (0.99, 1.46)	1.07 (0.89, 1.30)	0.82 (0.53, 1.25)	0.74 (0.55, 1.00)	0.78 (0.61, 1.01)	0.80 (0.67, 0.96)
Black, Non-Hispanic	953	**1.57 (1.25, 1.98)**	**1.55 (1.24, 1.93)**	**1.44 (1.16, 1.79)**	**1.37 (1.12, 1.68)**	0.68 (0.40, 1.16)	0.73 (0.53, 1.02)	0.89 (0.69, 1.16)	0.88 (0.72, 1.07)
Hispanic or Latino	695	1.37 (0.99, 1.89)	**1.44 (1.06, 1.95)**	**1.49 (1.12, 1.98)**	**1.40 (1.06, 1.85)**	0.58 (0.26, 1.29)	1.17 (0.77, 1.78)	1.15 (0.81, 1.64)	1.20 (0.93, 1.55)
White, Non-Hispanic	13,378	Ref	Ref	Ref	Ref	Ref	Ref	Ref	Ref
**Language**									
English	15,169	Ref	Ref	Ref	Ref	Ref	Ref	Ref	Ref
Non-English	1,306	0.93 (0.72, 1.21)	0.81 (0.63, 1.04)	0.83 (0.65, 1.05)	0.77 (0.61, 0.98)	0.84 (0.51, 1.39)	0.67 (0.47, 0.94)	0.70 (0.53, 0.93)	0.75 (0.61, 0.92)
**Sex**									
Female	5,926	0.81 (0.71, 0.93)	0.82 (0.72, 0.93)	0.82 (0.73, 0.92)	0.86 (0.77, 0.96)	0.96 (0.77, 1.19)	1.09 (0.95, 1.26)	1.04 (0.92, 1.17)	0.99 (0.90, 1.08)
Male	10,549	Ref	Ref	Ref	Ref	Ref	Ref	Ref	Ref

eTable 1 Legend. Cells include adjusted odds ratios and 95% confidence intervals (CIs). Cells are shaded in blue where entire 95% CI is estimated below 1. Cells are bolded where entire 95% CI is estimated above 1. Abbreviations: No., Number. Ref., Reference.

© 2026 Wang AZ et al. *JAMA Health Forum.*

eTable 2. Adjusted Probabilities of Completion of Cardiovascular Follow-Up Care Endpoints and Intermediate Steps

**Cardiology Referral Visits**			
**Outcome**	**Subgroup**	**Adjusted Probability, % (95% CI)**	**Risk Difference, % (vs Ref)**
Received Referral Order	Insurance: Commercial	19.4 (17.3, 21.5)	Ref
Received Referral Order	Insurance: Medicare Dual or Disabled	15.7 (13.2, 18.3)	-3.7
Received Referral Order	Insurance: Medicaid	14.7 (12.2, 17.2)	-4.7
Received Referral Order	Insurance: Medicare	25.0 (22.6, 27.3)	5.6
Received Referral Order	Race/Ethnicity: White, Non-Hispanic	21.8 (19.7, 23.9)	Ref
Received Referral Order	Race/Ethnicity: Asian, Non-Hispanic, and All Other	15.1 (12.5, 17.8)	-6.7
Received Referral Order	Race/Ethnicity: Black, Non-Hispanic	20.9 (17.4, 24.4)	-0.9
Received Referral Order	Race/Ethnicity: Hispanic or Latino	18.8 (15.0, 22.6)	-3.0
Received Referral Order	Sex: Male	20.9 (18.6, 23.1)	Ref
Received Referral Order	Sex: Female	21.4 (19.0, 23.8)	0.5
Received Referral Order	Language: English	21.5 (19.2, 23.7)	Ref
Received Referral Order	Language: Non-English	16.2 (13.0, 19.4)	-5.3
Scheduled an Ordered Referral	Insurance: Commercial	85.4 (80.7, 90.0)	Ref
Scheduled an Ordered Referral	Insurance: Medicare Dual or Disabled	80.5 (72.7, 88.3)	-4.9
Scheduled an Ordered Referral	Insurance: Medicaid	82.4 (74.8, 90.0)	-3.0
Scheduled an Ordered Referral	Insurance: Medicare	90.6 (87.5, 93.7)	5.2
Scheduled an Ordered Referral	Race/Ethnicity: White, Non-Hispanic	88.1 (84.3, 91.8)	Ref
Scheduled an Ordered Referral	Race/Ethnicity: Asian, Non-Hispanic, and All Other	87.4 (81.0, 93.8)	-0.7
Scheduled an Ordered Referral	Race/Ethnicity: Black, Non-Hispanic	73.9 (64.2, 83.7)	-14.2
Scheduled an Ordered Referral	Race/Ethnicity: Hispanic or Latino	80.9 (71.3, 90.6)	-7.2
Scheduled an Ordered Referral	Sex: Male	87.0 (82.6, 91.4)	Ref
Scheduled an Ordered Referral	Sex: Female	86.8 (81.9, 91.7)	-0.2
Scheduled an Ordered Referral	Language: English	87.1 (82.8, 91.4)	Ref
Scheduled an Ordered Referral	Language: Non-English	85.3 (77.5, 93.2)	-1.8

© 2026 Wang AZ et al. *JAMA Health Forum.*

eTable 2 (Continued). Adjusted Probabilities of Completion of Cardiovascular Follow-Up Care Endpoints and Intermediate Steps

Cardiology Referral Visits			
**Outcome**	**Subgroup**	**Adjusted Probability, % (95% CI)**	**Risk Difference, % (vs Ref)**
Completed a Scheduled Visit	Insurance: Commercial	94.0 (90.7, 97.3)	Ref
Completed a Scheduled Visit	Insurance: Medicare Dual or Disabled	92.6 (86.9, 98.2)	-1.4
Completed a Scheduled Visit	Insurance: Medicaid	90.6 (83.6, 97.6)	-3.4
Completed a Scheduled Visit	Insurance: Medicare	94.9 (92.3, 97.4)	0.9
Completed a Scheduled Visit	Race/Ethnicity: White, Non-Hispanic	94.0 (90.9, 97.1)	Ref
Completed a Scheduled Visit	Race/Ethnicity: Asian, Non-Hispanic, and All Other	93.9 (88.8, 99.0)	-0.1
Completed a Scheduled Visit	Race/Ethnicity: Black, Non-Hispanic	93.9 (88.4, 99.4)	-0.1
Completed a Scheduled Visit	Race/Ethnicity: Hispanic or Latino	91.5 (83.5, 99.5)	-2.5
Completed a Scheduled Visit	Sex: Male	94.1 (90.6, 97.5)	Ref
Completed a Scheduled Visit	Sex: Female	93.5 (89.6, 97.5)	-0.6
Completed a Scheduled Visit	Language: English	93.7 (90.2, 97.2)	Ref
Completed a Scheduled Visit	Language: Non-English	95.6 (90.9, 100.3)	1.9
Overall: Completed Visit (Unconditional)	Insurance: Commercial	16.0 (14.0, 17.9)	Ref
Overall: Completed Visit (Unconditional)	Insurance: Medicare Dual or Disabled	12.0 (9.8, 14.3)	-4.0
Overall: Completed Visit (Unconditional)	Insurance: Medicaid	11.3 (9.1, 13.6)	-4.7
Overall: Completed Visit (Unconditional)	Insurance: Medicare	21.7 (19.4, 23.9)	5.7
Overall: Completed Visit (Unconditional)	Race/Ethnicity: White, Non-Hispanic	18.5 (16.6, 20.5)	Ref
Overall: Completed Visit (Unconditional)	Race/Ethnicity: Asian, Non-Hispanic, and All Other	12.7 (10.3, 15.1)	-5.8
Overall: Completed Visit (Unconditional)	Race/Ethnicity: Black, Non-Hispanic	15.2 (12.2, 18.1)	-3.3
Overall: Completed Visit (Unconditional)	Race/Ethnicity: Hispanic or Latino	14.2 (10.9, 17.6)	-4.3
Overall: Completed Visit (Unconditional)	Sex: Male	17.6 (15.5, 19.7)	Ref
Overall: Completed Visit (Unconditional)	Sex: Female	17.9 (15.6, 20.1)	0.3
Overall: Completed Visit (Unconditional)	Language: English	18.0 (16.0, 20.1)	Ref
Overall: Completed Visit (Unconditional)	Language: Non-English	13.4 (10.5, 16.4)	-4.6

© 2026 Wang AZ et al. *JAMA Health Forum.*

eTable 2 (Continued). Adjusted Probabilities of Completion of Cardiovascular Follow-Up Care Endpoints and Intermediate Steps

Coronary Artery Disease (CAD) Testing			
**Outcome**	**Subgroup**	**Adjusted Probability, % (95% CI)**	**Risk Difference, % (vs Ref)**
Received Test Order	Insurance: Commercial	17.1 (14.9, 19.3)	Ref
Received Test Order	Insurance: Medicare Dual or Disabled	9.2 (7.1, 11.3)	-7.9
Received Test Order	Insurance: Medicaid	13.0 (10.4, 15.6)	-4.1
Received Test Order	Insurance: Medicare	12.5 (10.8, 14.3)	-4.6
Received Test Order	Race/Ethnicity: White, Non-Hispanic	13.5 (11.7, 15.3)	Ref
Received Test Order	Race/Ethnicity: Asian, Non-Hispanic, and All Other	13.7 (11.0, 16.4)	0.2
Received Test Order	Race/Ethnicity: Black, Non-Hispanic	17.0 (13.6, 20.5)	3.5
Received Test Order	Race/Ethnicity: Hispanic or Latino	14.4 (10.8, 18.0)	0.9
Received Test Order	Sex: Male	14.5 (12.4, 16.6)	Ref
Received Test Order	Sex: Female	12.4 (10.4, 14.4)	-2.1
Received Test Order	Language: English	13.9 (11.9, 15.9)	Ref
Received Test Order	Language: Non-English	11.6 (8.7, 14.5)	-2.3
Scheduled an Ordered Test	Insurance: Commercial	90.6 (86.6, 94.5)	Ref
Scheduled an Ordered Test	Insurance: Medicare Dual or Disabled	84.1 (75.3, 93.0)	-6.5
Scheduled an Ordered Test	Insurance: Medicaid	89.4 (82.7, 96.0)	-1.2
Scheduled an Ordered Test	Insurance: Medicare	87.4 (82.3, 92.5)	-3.2
Scheduled an Ordered Test	Race/Ethnicity: White, Non-Hispanic	88.4 (83.7, 93.0)	Ref
Scheduled an Ordered Test	Race/Ethnicity: Asian, Non-Hispanic, and All Other	89.3 (82.9, 95.8)	0.9
Scheduled an Ordered Test	Race/Ethnicity: Black, Non-Hispanic	89.4 (82.5, 96.3)	1.0
Scheduled an Ordered Test	Race/Ethnicity: Hispanic or Latino	91.9 (84.8, 98.9)	3.5
Scheduled an Ordered Test	Sex: Male	89.5 (85.0, 94.1)	Ref
Scheduled an Ordered Test	Sex: Female	86.9 (80.8, 92.9)	-2.6
Scheduled an Ordered Test	Language: English	88.8 (84.0, 93.6)	Ref
Scheduled an Ordered Test	Language: Non-English	87.2 (78.2, 96.3)	-1.6

© 2026 Wang AZ et al. *JAMA Health Forum.*

eTable 2 (Continued). Adjusted Probabilities of Completion of Cardiovascular Follow-Up Care Endpoints and Intermediate Steps

Coronary Artery Disease (CAD) Testing			
**Outcome**	**Subgroup**	**Adjusted Probability, % (95% CI)**	**Risk Difference, % (vs Ref)**
Completed a Scheduled Test	Insurance: Commercial	95.9 (93.0, 98.7)	Ref
Completed a Scheduled Test	Insurance: Medicare Dual or Disabled	92.9 (85.9, 100.0)	-3.0
Completed a Scheduled Test	Insurance: Medicaid	97.4 (94.1, 100.7)	1.5
Completed a Scheduled Test	Insurance: Medicare	95.3 (91.8, 98.7)	-0.6
Completed a Scheduled Test	Race/Ethnicity: White, Non-Hispanic	95.0 (91.5, 98.6)	Ref
Completed a Scheduled Test	Race/Ethnicity: Asian, Non-Hispanic, and All Other	98.7 (96.7, 100.8)	3.7
Completed a Scheduled Test	Race/Ethnicity: Black, Non-Hispanic	97.6 (94.3, 101.0)	2.6
Completed a Scheduled Test	Race/Ethnicity: Hispanic or Latino	NA	NA
Completed a Scheduled Test	Sex: Male	95.6 (92.4, 98.9)	Ref
Completed a Scheduled Test	Sex: Female	95.4 (91.6, 99.2)	-0.2
Completed a Scheduled Test	Language: English	95.6 (92.3, 98.9)	Ref
Completed a Scheduled Test	Language: Non-English	95.8 (89.7, 101.8)	0.2
Overall: Completed Test (Unconditional)	Insurance: Commercial	14.9 (12.8, 17.0)	Ref
Overall: Completed Test (Unconditional)	Insurance: Medicare Dual or Disabled	7.2 (5.4, 9.1)	-7.7
Overall: Completed Test (Unconditional)	Insurance: Medicaid	11.4 (8.9, 13.9)	-3.5
Overall: Completed Test (Unconditional)	Insurance: Medicare	10.4 (8.8, 12.1)	-4.5
Overall: Completed Test (Unconditional)	Race/Ethnicity: White, Non-Hispanic	11.3 (9.7, 13.0)	Ref
Overall: Completed Test (Unconditional)	Race/Ethnicity: Asian, Non-Hispanic, and All Other	12.1 (9.5, 14.7)	0.8
Overall: Completed Test (Unconditional)	Race/Ethnicity: Black, Non-Hispanic	14.9 (11.6, 18.1)	3.6
Overall: Completed Test (Unconditional)	Race/Ethnicity: Hispanic or Latino	13.2 (9.7, 16.8)	1.9
Overall: Completed Test (Unconditional)	Sex: Male	12.4 (10.5, 14.4)	Ref
Overall: Completed Test (Unconditional)	Sex: Female	10.3 (8.5, 12.1)	-2.1
Overall: Completed Test (Unconditional)	Language: English	11.8 (10.0, 13.6)	Ref
Overall: Completed Test (Unconditional)	Language: Non-English	9.9 (7.2, 12.6)	-1.9

eTable 2 Legend. Adjusted predicted probabilities (%) and 95% confidence intervals were estimated from fully adjusted logistic regression models corresponding to the adjusted odds ratios presented in the main text. Predicted probabilities were obtained using marginal standardization over the observed covariate distribution within each subgroup.

© 2026 Wang AZ et al. *JAMA Health Forum*.

eTable 3. E-values for Adjusted Associations Between Key Covariates and Coronary Artery Disease Testing and Cardiology Referral Outcomes

**Coronary Artery Disease (CAD) Testing**		
**Outcome: Received Test Order**		
**Subgroup**	**aOR (95% CI)**	**E-Value**
Medicare	0.66 (0.58, 0.77)	1.75
Medicaid	0.74 (0.62, 0.88)	1.60
Medicare Dual or Disabled	0.48 (0.39, 0.59)	2.24
Black, Non-Hispanic	1.32 (1.09, 1.61)	1.57
Non-English	0.80 (0.64, 1.00)	1.48
Female	0.88 (0.80, 0.98)	1.32
**Outcome: Overall - Completed CAD Testing (Unconditional)**		
Medicare	0.66 (0.57, 0.77)	1.76
Medicaid	0.74 (0.62, 0.89)	1.59
Medicare Dual or Disabled	0.45 (0.36, 0.56)	2.36
Black, Non-Hispanic	1.37 (1.12, 1.68)	1.62
Hispanic or Latino	1.40 (1.06, 1.85)	1.65
Non-English	0.77 (0.61, 0.98)	1.53
Female	0.86 (0.77, 0.96)	1.37

© 2026 Wang AZ et al. *JAMA Health Forum*.

eTable 3 (Continued). E-values for Adjusted Associations Between Key Covariates and Coronary Artery Disease Testing and Cardiology Referral Outcomes

**Cardiology Referral Visits**		
**Subgroup**	**aOR (95% CI)**	**E-Value**
**Outcome: Received Referral Order**		
Medicaid	0.68 (0.58, 0.80)	1.72
Medicare Dual or Disabled	0.53 (0.45, 0.63)	2.09
Asian, Non-Hispanic	0.79 (0.67, 0.94)	1.49
Hispanic or Latino	1.33 (1.06, 1.69)	1.58
Non-English	0.72 (0.60, 0.87)	1.64
**Outcome: Scheduled an Ordered Referral**		
Medicare Dual or Disabled	0.52 (0.35, 0.77)	2.13
Black, Non-Hispanic	0.45 (0.31, 0.65)	2.35
**Outcome: Overall - Completed Visit (Unconditional)**		
Medicaid	0.66 (0.55, 0.79)	1.76
Medicare Dual or Disabled	0.47 (0.39, 0.57)	2.27
Asian, Non-Hispanic	0.80 (0.67, 0.96)	1.48
Non-English	0.75 (0.61, 0.92)	1.57

eTable 3 Legend. E-values quantify the minimum strength of association an unmeasured confounder would need with both exposure and outcome to explain away the observed adjusted odds ratio. Presented are E-values for statistically significant associations (95% confidence intervals excluding 1) of insurance type, race/ethnicity, language, and sex across coronary artery disease testing and cardiology referral outcomes. Larger E-values indicate greater robustness to unmeasured confounding.

© 2026 Wang AZ et al. *JAMA Health Forum*.

eTable 4. Adjusted Odds Ratios for Referral Order by Subgroup Using IPCW-Weighted and Unweighted Logistic Regression Models

Model Outcome	Subgroup	IPCW aOR (95% CI)	IPCW P-Value	Unweighted aOR (95% CI)	Unweighted P-Value
Referral Order	Sex: Female	1.010 (0.929, 1.100)	0.78	1.010 (0.927, 1.100)	0.82
Referral Order	Insurance: Medicaid	0.681 (0.580, 0.800)	<0.005	0.682 (0.581, 0.801)	<0.005
Referral Order	Insurance: Medicare	0.905 (0.802, 1.020)	0.10	0.904 (0.801, 1.020)	0.10
Referral Order	Insurance: Medicare Dual or Disabled	0.531 (0.451, 0.625)	<0.005	0.531 (0.451, 0.625)	<0.005
Referral Order	Language: Non-English	0.720 (0.598, 0.868)	<0.005	0.719 (0.597, 0.866)	<0.005
Referral Order	Race/Ethnicity: Asian & Other	0.792 (0.671, 0.935)	0.01	0.794 (0.672, 0.937)	0.01
Referral Order	Race/Ethnicity: Black	1.050 (0.876, 1.250)	0.62	1.050 (0.876, 1.250)	0.62
Referral Order	Race/Ethnicity: Hispanic	1.330 (1.050, 1.690)	0.02	1.330 (1.060, 1.690)	0.02
Scheduled Referral	Sex: Female	0.877 (0.692, 1.110)	0.28	0.877 (0.691, 1.110)	0.28
Scheduled Referral	Insurance: Medicaid	0.968 (0.641, 1.460)	0.88	0.969 (0.643, 1.460)	0.88
Scheduled Referral	Insurance: Medicare	0.899 (0.646, 1.250)	0.53	0.899 (0.645, 1.250)	0.53
Scheduled Referral	Insurance: Medicare Dual or Disabled	0.514 (0.344, 0.770)	<0.005	0.517 (0.345, 0.774)	<0.005
Scheduled Referral	Language: Non-English	0.932 (0.571, 1.520)	0.78	0.935 (0.573, 1.530)	0.79
Scheduled Referral	Race/Ethnicity: Asian & Other	1.140 (0.713, 1.830)	0.58	1.140 (0.713, 1.820)	0.58
Scheduled Referral	Race/Ethnicity: Black	0.448 (0.306, 0.655)	<0.005	0.448 (0.306, 0.654)	<0.005
Scheduled Referral	Race/Ethnicity: Hispanic	0.798 (0.456, 1.400)	0.43	0.795 (0.455, 1.390)	0.42
Referral Visit Completion (Conditional)	Sex: Female	0.797 (0.557, 1.140)	0.22	0.798 (0.558, 1.140)	0.22
Referral Visit Completion (Conditional)	Insurance: Medicaid	0.591 (0.316, 1.100)	0.10	0.593 (0.318, 1.110)	0.10
Referral Visit Completion (Conditional)	Insurance: Medicare	0.779 (0.467, 1.300)	0.34	0.777 (0.466, 1.290)	0.33
Referral Visit Completion (Conditional)	Insurance: Medicare Dual or Disabled	0.530 (0.271, 1.040)	0.06	0.530 (0.271, 1.040)	0.06
Referral Visit Completion (Conditional)	Language: Non-English	1.980 (0.777, 5.050)	0.15	1.980 (0.778, 5.040)	0.15
Referral Visit Completion (Conditional)	Race/Ethnicity: Asian & Other	0.986 (0.494, 1.970)	0.97	0.984 (0.494, 1.960)	0.96
Referral Visit Completion (Conditional)	Race/Ethnicity: Black	1.030 (0.457, 2.310)	0.95	1.030 (0.459, 2.310)	0.94
Referral Visit Completion (Conditional)	Race/Ethnicity: Hispanic	0.682 (0.277, 1.680)	0.41	0.681 (0.277, 1.670)	0.40

© 2026 Wang AZ et al. *JAMA Health Forum.*

Model Outcome	Subgroup	IPCW aOR (95% CI)	IPCW P-Value	Unweighted aOR (95% CI)	Unweighted P-Value
Referral Visit Completion (Unconditional)	Sex: Female	0.988 (0.902, 1.080)	0.80	0.986 (0.899, 1.080)	0.76
Referral Visit Completion (Unconditional)	Insurance: Medicaid	0.660 (0.553, 0.788)	<0.005	0.661 (0.554, 0.789)	<0.005
Referral Visit Completion (Unconditional)	Insurance: Medicare	0.884 (0.778, 1.010)	0.06	0.883 (0.776, 1.000)	0.06
Referral Visit Completion (Unconditional)	Insurance: Medicare Dual or Disabled	0.472 (0.394, 0.565)	<0.005	0.472 (0.395, 0.566)	<0.005
Referral Visit Completion (Unconditional)	Language: Non-English	0.752 (0.616, 0.919)	0.01	0.752 (0.615, 0.918)	0.01
Referral Visit Completion (Unconditional)	Race/Ethnicity: Asian & Other	0.801 (0.670, 0.958)	0.02	0.802 (0.671, 0.959)	0.02
Referral Visit Completion (Unconditional)	Race/Ethnicity: Black	0.878 (0.720, 1.070)	0.20	0.878 (0.720, 1.070)	0.20
Referral Visit Completion (Unconditional)	Race/Ethnicity: Hispanic	1.200 (0.925, 1.550)	0.17	1.200 (0.925, 1.550)	0.17
Referral Order	Sex: Female	0.950 (0.850, 1.060)	0.35	0.950 (0.850, 1.060)	0.36
Referral Order	Insurance: Medicaid	0.700 (0.580, 0.850)	<0.005	0.700 (0.580, 0.850)	<0.005
Referral Order	Insurance: Medicare	0.890 (0.770, 1.020)	0.10	0.890 (0.770, 1.020)	0.09
Referral Order	Insurance: Medicare Dual or Disabled	0.550 (0.450, 0.660)	<0.005	0.550 (0.450, 0.660)	<0.005
Referral Order	Language: Non-English	0.750 (0.610, 0.910)	<0.005	0.750 (0.610, 0.910)	<0.005
Referral Order	Race/Ethnicity: Asian & Other	0.810 (0.680, 0.960)	0.02	0.810 (0.680, 0.960)	0.02
Referral Order	Race/Ethnicity: Black	1.000 (0.830, 1.200)	0.99	1.000 (0.830, 1.200)	0.98
Referral Order	Race/Ethnicity: Hispanic	1.220 (0.960, 1.540)	0.10	1.220 (0.950, 1.540)	0.11
Scheduled Referral	Sex: Female	0.910 (0.700, 1.180)	0.46	0.910 (0.690, 1.190)	0.47
Scheduled Referral	Insurance: Medicaid	1.020 (0.670, 1.550)	0.93	1.020 (0.680, 1.540)	0.94
Scheduled Referral	Insurance: Medicare	0.890 (0.640, 1.240)	0.49	0.890 (0.640, 1.240)	0.49
Scheduled Referral	Insurance: Medicare Dual or Disabled	0.480 (0.310, 0.750)	<0.005	0.490 (0.310, 0.760)	<0.005
Scheduled Referral	Language: Non-English	0.930 (0.570, 1.520)	0.78	0.930 (0.570, 1.530)	0.79
Scheduled Referral	Race/Ethnicity: Asian & Other	1.130 (0.690, 1.850)	0.63	1.130 (0.700, 1.830)	0.62
Scheduled Referral	Race/Ethnicity: Black	0.450 (0.310, 0.660)	<0.005	0.450 (0.310, 0.660)	<0.005
Scheduled Referral	Race/Ethnicity: Hispanic	0.790 (0.450, 1.390)	0.42	0.790 (0.450, 1.380)	0.42
Referral Visit Completion (Conditional)	Sex: Female	0.850 (0.570, 1.260)	0.41	0.850 (0.580, 1.260)	0.42
Referral Visit Completion (Conditional)	Insurance: Medicaid	0.620 (0.330, 1.140)	0.12	0.630 (0.340, 1.150)	0.13

© 2026 Wang AZ et al. *JAMA Health Forum*.

Model Outcome	Subgroup	IPCW aOR (95% CI)	IPCW P-Value	Unweighted aOR (95% CI)	Unweighted P-Value
Referral Visit Completion (Conditional)	Insurance: Medicare	0.740 (0.430, 1.280)	0.29	0.740 (0.430, 1.270)	0.28
Referral Visit Completion (Conditional)	Insurance: Medicare Dual or Disabled	0.480 (0.230, 1.000)	0.05	0.480 (0.230, 1.000)	0.05
Referral Visit Completion (Conditional)	Language: Non-English	2.100 (0.790, 5.570)	0.14	2.110 (0.780, 5.640)	0.14
Referral Visit Completion (Conditional)	Race/Ethnicity: Asian & Other	1.020 (0.470, 2.240)	0.96	1.020 (0.470, 2.230)	0.95
Referral Visit Completion (Conditional)	Race/Ethnicity: Black	1.050 (0.440, 2.500)	0.91	1.050 (0.450, 2.500)	0.91
Referral Visit Completion (Conditional)	Race/Ethnicity: Hispanic	0.700 (0.280, 1.720)	0.44	0.700 (0.280, 1.710)	0.44
Referral Visit Completion (Unconditional)	Sex: Female	0.980 (0.900, 1.070)	0.65	0.980 (0.890, 1.060)	0.66
Referral Visit Completion (Unconditional)	Insurance: Medicaid	0.670 (0.550, 0.810)	<0.005	0.670 (0.550, 0.810)	<0.005
Referral Visit Completion (Unconditional)	Insurance: Medicare	0.890 (0.770, 1.020)	0.08	0.890 (0.770, 1.020)	0.08
Referral Visit Completion (Unconditional)	Insurance: Medicare Dual or Disabled	0.470 (0.390, 0.560)	<0.005	0.470 (0.390, 0.560)	<0.005
Referral Visit Completion (Unconditional)	Language: Non-English	0.750 (0.620, 0.920)	0.01	0.750 (0.620, 0.920)	0.01
Referral Visit Completion (Unconditional)	Race/Ethnicity: Asian & Other	0.800 (0.670, 0.960)	0.02	0.800 (0.670, 0.960)	0.02
Referral Visit Completion (Unconditional)	Race/Ethnicity: Black	0.880 (0.720, 1.070)	0.20	0.880 (0.720, 1.070)	0.20
Referral Visit Completion (Unconditional)	Race/Ethnicity: Hispanic	1.190 (0.930, 1.540)	0.17	1.190 (0.930, 1.540)	0.17

eTable 4 Legend. Adjusted odds ratios (OR) with 95% confidence intervals (CI) and p-values for the association between the exposure/variable and outcome, stratified by subgroup. Estimates are shown for both inverse probability of censoring weighted (IPCW) and unweighted logistic regression models. IPCW weighting accounts for potential informative censoring. Subgroups with odds ratios below 1 indicate lower odds of referral order compared to the reference group. Statistically significant results ($p < 0.05$) are indicated in the table.

© 2026 Wang AZ et al. *JAMA Health Forum.*

eTable 5. Emergency Department (ED) Site-Level Heterogeneity via Random Intercept Mixed-Effects Models

Outcome	Number of Patients	Number of ED Sites	ED Random Intercept Variance	Intraclass Correlation (ICC)	Between-Site Heterogeneity (OR Range)
CAD Testing Completion	16,475	10	0.047	1.4%	0.84-1.38
Referral Visit Completion	16,475	10	0.055	1.6%	0.68-1.25

eTable 5 Legend. Site-level heterogeneity for referral and CAD testing completion was estimated using random intercept mixed-effects logistic regression. Variance reflects between-ED differences; ICC shows the proportion of total variance due to ED clustering. The OR range indicates variation in baseline odds across EDs after adjustment. Abbreviations: ED, emergency department; OR, odds ratio.

© 2026 Wang AZ et al. *JAMA Health Forum*.

eTable 6. Time to Completion of Cascade Steps

Outcome	Subgroup	Mean Days	Median (IQR), Days
**CAD Testing Completion**	Insurance: Commercial	29.5	9 (1-43)
	Insurance: Medicaid	29.0	5 (1-43)
	Insurance: Medicare	33.5	6 (1-47.8)
	Insurance: Medicare Dual/Disabled	39.4	5 (1-72)
	Race/Ethnicity: White, NH	33.6	8 (1-50)
	Race/Ethnicity: Asian & Other	24.3	4 (1-28)
	Race/Ethnicity: Black, NH	23.1	4 (1-21.8)
	Race/Ethnicity: Hispanic	29.4	5 (1-44)
	Sex: Male	31.5	7 (1-47)
	Sex: Female	32.4	7 (1-47)
	Language: English	32.0	7 (1-47)
	Language: Non-English	29.4	3 (1-38.5)
**Referral Visit Completion**	Insurance: Commercial	58.5	45 (23-87)
	Insurance: Medicaid	58.9	42.5 (22-87.5)
	Insurance: Medicare	61.9	46 (23-92)
	Insurance: Medicare Dual/Disabled	65.0	54 (23-94.5)
	Race/Ethnicity: White, NH	61.2	46 (22-91)
	Race/Ethnicity: Asian & Other	57.6	46 (24-81)
	Race/Ethnicity: Black, NH	58.3	43.5 (25-81)
	Race/Ethnicity: Hispanic	62.7	51.5 (23-91)
	Sex: Male	60.8	46 (23-91)
	Sex: Female	61.1	45 (23-90.3)
	Language: English	60.9	46 (22-91)
	Language: Non-English	61.4	51 (25-86)

eTable 6 Legend. Values represent time from ED visit to completion among patients who completed each step. Differences across subgroups were assessed using Kruskal-Wallis tests (all $p > 0.38$ for referral visit completion; $p = 0.03$ for CAD testing by race/ethnicity; all other $p > 0.07$ for CAD testing completion). Abbreviations: NH, Non-Hispanic.

© 2026 Wang AZ et al. *JAMA Health Forum*.

eTable 7. Adjusted Completion Rates of Primary Care Office Visits within Six Months After an Index ED Visit

		Primary Care Office Visits	
**Subgroup**	No. of Patients N=16,475	**Unadjusted Completion Rate (%)**	**Adjusted Odds Ratio for Completion (95% CI)**
**Insurance Payer**			
Commercial	5,299	54.3	Ref
Medicare	7,480	54.2	0.94 (0.85, 1.04)
Medicaid	1,759	45.7	0.75 (0.66, 0.84)
Medicare Dual or Disabled	1,773	45.5	0.69 (0.61, 0.78)
**Race/Ethnicity**			
Asian, Non-Hispanic	1,449	53.9	1.12 (0.99, 1.26)
Black, Non-Hispanic	953	51.2	**1.16 (1.00, 1.33)**
Hispanic or Latino	695	49.9	1.07 (0.89, 1.28)
White, Non-Hispanic	13,378	52.3	Ref
**Language**			
English	15,169	52.4	Ref
Non-English	1,306	51.1	1.01 (0.88, 1.16)
**Sex**			
Female	5,926	52.6	0.99 (0.93, 1.06)
Male	10,549	52.0	Ref

eTable 7 Legend. Cells include adjusted odds ratio point estimates and 95% confidence intervals (CIs). Cells are shaded in blue where 95% CI is estimated below 1 and does not overlap with 1. Cells are bolded where 95% CI is estimated above 1 and does not overlap with 1. Abbreviations: No., Number. Ref., Reference.

© 2026 Wang AZ et al. *JAMA Health Forum*.

eTable 8. Unadjusted Completion Rates of Procedures for Coronary Artery Disease within Six Months After an Index ED Visit

		**Completed Interventions, %**	
**Subgroup**	Number of Patients	**ICA**	**CABG (Surgery)**
**All Patients**	16,475	5.6	0.7
**Insurance Payer**			
Commercial	5,299	5.6	0.6
Medicare	7,480	6.1	0.8
Medicaid	1,759	4.7	0.5
Medicare Dual or Disabled	1,773	4.5	0.6
**Race**			
Asian and All Other, Non-Hispanic	1,449	3.5	0.7
Black, Non-Hispanic	953	5.0	0.5
Hispanic or Latino	695	7.1	1.0
White, Non-Hispanic	13,378	5.8	0.6
**Language**			
English	15,169	5.5	0.6
Non-English	1,306	5.8	0.8
**Sex**			
Female	5,926	4.9	0.3
Male	10,549	5.9	0.9

eTable 8 Legend. Abbreviations: ICA, invasive coronary angiography; CABG, coronary artery bypass graft.

© 2026 Wang AZ et al. *JAMA Health Forum*.

eTable 9. Adjusted Completion Rates of Procedures for Coronary Artery Disease within Six Months After an Index ED Visit

		Completed Interventions	
**Subgroup**	Number of Patients N=16,475	**ICA**	**CABG (Surgery)**
**Insurance Payer**			
Commercial	5,299	Ref	Ref
Medicare	7,480	0.77 (0.62, 0.96)	1.10 (0.60, 2.02)
Medicaid	1,759	0.52 (0.39, 0.70)	0.63 (0.28, 1.42)
Medicare Dual or Disabled	1,773	0.43 (0.32, 0.58)	0.68 (0.30, 1.52)
**Race/Ethnicity**			
Asian, Non-Hispanic	1,449	0.63 (0.45, 0.89)	1.36 (0.65, 2.85)
Black, Non-Hispanic	953	0.69 (0.49, 0.97)	0.62 (0.22, 1.74)
Hispanic or Latino	695	1.27 (0.85, 1.91)	1.76 (0.67, 4.63)
White, Non-Hispanic	13,378	Ref	Ref
**Language**			
English	15,169	Ref	Ref
Non-English	1,306	1.06 (0.77, 1.45)	0.87(0.38, 1.95)
**Sex**			
Female	5,926	0.91 (0.77, 1.08)	0.33 (0.19, 0.57)
Male	10,549	Ref	Ref

eTable 9 Legend. Cells include adjusted odds ratio point estimates and 95% confidence intervals (CIs). Cells are shaded in blue where 95% CI is estimated below 1 and does not overlap with 1. Cells are bolded where 95% CI is estimated above 1 and does not overlap with 1. Abbreviations: No., Number; Ref., Reference; ICA, invasive coronary angiography; CABG, coronary artery bypass graft.

© 2026 Wang AZ et al. *JAMA Health Forum*.

References in Supplementary Material

1. Gulati M, Levy PD, Mukherjee D, et al. 2021 AHA/ACC/ASE/CHEST/SAEM/SCCT/SCMR Guideline for the Evaluation and Diagnosis of Chest Pain: A Report of the American College of Cardiology/American Heart Association Joint Committee on Clinical Practice Guidelines. *Circulation*. 2021;144(22). doi:10.1161/CIR.0000000000001029

2. Backus BE, Six AJ, Kelder JC, et al. A prospective validation of the HEART score for chest pain patients at the emergency department. *International Journal of Cardiology*. 2013;168(3):2153-2158. doi:10.1016/j.ijcard.2013.01.255

3. Mahler SA, Riley RF, Hiestand BC, et al. The HEART Pathway Randomized Trial: Identifying Emergency Department Patients With Acute Chest Pain for Early Discharge. *Circ: Cardiovascular Quality and Outcomes*. 2015;8(2):195-203. doi:10.1161/CIRCOUTCOMES.114.001384

4. Ouyang D, Theurer J, Stein NR, et al. Electrocardiographic deep learning for predicting post-procedural mortality: a model development and validation study. *The Lancet Digital Health*. 2024;6(1):e70-e78. doi:10.1016/S2589-7500(23)00220-0

5. Steingrimsson JA, Gatsonis C, Li B, Dahabreh IJ. Transporting a Prediction Model for Use in a New Target Population. *American Journal of Epidemiology*. 2023;192(2):296-304. doi:10.1093/aje/kwac128

6. Sugiyama M, Krauledat M, Müller KR. Covariate Shift Adaptation by Importance Weighted Cross Validation. *Journal of Machine Learning Research*. 2007;8:985-1005.

7. VanderWeele TJ, Ding P. Sensitivity Analysis in Observational Research: Introducing the E-Value. *Ann Intern Med*. 2017;167(4):268-274. doi:10.7326/M16-2607

© 2026 Wang AZ et al. *JAMA Health Forum*.
