# Supplementary material for: Sources of Variation in Cardiovascular Care Cascades
Source: JAMA Health Forum. 2026 Apr 3;7(4):e260491. doi: 10.1001/jamahealthforum.2026.0491 (PMC13049495; doi:10.1001/jamahealthforum.2026.0491)
Supplement: Supplement 2. — Data Sharing Statement [file jamahealthforum-e260491-s002.pdf]

Data Sharing Statement

Wang. Sources of Variation in Cardiovascular Care Cascades. *JAMA Health Forum*. Published April 03, 2026. doi:10.1001/jamahealthforum.2026.0491

Data
Data available: No

Additional information: The data are from confidential electronic health records and cannot be publicly shared.
